# Deubiquitylation Machinery Is Required for Embryonic Polarity in *Caenorhabditis elegans*

**DOI:** 10.1371/journal.pgen.1003092

**Published:** 2012-11-29

**Authors:** Richard J. McCloskey, Kenneth J. Kemphues

**Affiliations:** Department of Molecular Biology and Genetics, Cornell University, Ithaca, New York, United States of America; Harvard University, United States of America

## Abstract

The *Caenorhabditis elegans* one-cell embryo polarizes in response to a cue from the paternally donated centrosome and asymmetrically segregates cell fate determinants that direct the developmental program of the worm. We have found that genes encoding putative deubiquitylating enzymes (DUBs) are required for polarization of one-cell embryos. Maternal loss of the proteins MATH-33 and USP-47 leads to variable inability to correctly establish and maintain asymmetry as defined by posterior and anterior polarity proteins PAR-2 and PAR-3. The first observable defect is variable positioning of the centrosome with respect to the cell cortex and the male pronucleus. The severity of the polarity defects correlates with distance of the centrosome from the cortex. Furthermore, polarity defects can be bypassed by mutations that bring the centrosome in close proximity to the cortex. In addition we find that polarity and centrosome positioning defects can be suppressed by compromising protein turnover. We propose that the DUB activity of MATH-33 and USP-47 stabilizes one or more proteins required for association of the centrosome with the cortex. Because these DUBs are homologous to two members of a group of DUBs that act in fission yeast polarity, we tested additional members of that family and found that another *C. elegans* DUB gene, *usp-46*, also contributes to polarity. Our finding that deubiquitylating enzymes required for polarity in *Schizosaccharomyces pombe* are also required in *C. elegans* raises the possibility that these DUBs act through an evolutionarily conserved mechanism to control cell polarity.

## Author Summary

In eukaryotes, modification of proteins by the covalent ligation of a protein called ubiquitin is an important regulatory mechanism. In this study we found that deubiquitylation enzymes, which are known to cleave ubiquitin off of target proteins, are required for asymmetry in one-cell embryos of the nematode *C. elegans*. In one-cell embryos the establishment of asymmetry depends on a signal from the centrosome, a microtubule-organizing center. This signal breaks homogeneity in the contractile cytoskeleton located at the cortex of the embryo. We have identified three deubiquitylation enzymes that are necessary for the centrosome to properly localize adjacent to the cortex to perform its symmetry-breaking role. Furthermore, a homologous group of enzymes in fission yeast also regulates cell polarity. Our results suggest that a novel mechanism of centrosome localization regulated by ubiquitylation exists in *C. elegans*; this mechanism is masked by genetic redundancy and may be an evolutionarily conserved mechanism for cell asymmetry.

## Introduction

Asymmetric cell division is a key mechanism for generating cellular diversity during development. The embryo of the nematode *Caenorhabditis elegans* is an excellent model for studying this mechanism. Much of the cellular machinery that controls the process of asymmetry in vertebrate organisms is conserved in the nematode. In particular, the *par* genes, which were discovered in *C. elegans*, are part of a conserved cassette of key polarity regulators [1], [2]. In *C. elegans* one-cell embryos, a group of physically interacting proteins, PAR-3, PAR-6, and PKC-3, initially distribute uniformly around the cell cortex but then polarize into what is defined as the anterior domain [3]. At the same time, PAR-1, PAR-2, and LGL-1 become enriched at the posterior cortex [4]–[7]. These two PAR domains in the anterior and posterior remain mutually exclusive in an inter-dependent fashion throughout the cell cycle, and are partitioned asymmetrically into the two daughter cells, AB and P1, which are also asymmetric in their size and cell fate determinant composition [3], [8].

The establishment of distinct PAR domains occurs through an interaction of the centrosome, nucleated by centrioles provided by the sperm, with the cortex [9]. This interaction breaks the symmetry of the embryo and specifies the posterior pole [10]–[13]. This break in symmetry is mediated primarily by a local down-regulation of the small GTPase RHO-1, leading to local inactivation of the actomyosin cytoskeleton [12], [14]–[16], and through a redundant microtubule-mediated recruitment of PAR-2 to the cortex [17], [18]. The local inactivation of cytoskeletal contractility causes an actomyosin cortical flow towards the anterior, and the proteins PAR-3, PAR-6, and PKC-3 move with the cortical flow, leaving behind an unoccupied cortex to which the posterior PARs are recruited [12], [19]. Mutations that block centrosome maturation also block the cortical flow and polarity establishment [20]–[22]. In addition, mutation of the gene *pam-1* reduces the association time of centrosomes at the cortex and also blocks polarity establishment [23], [24].

Establishment is completed by prophase of the first mitosis. The anterior-posterior PAR domains are maintained through the rest of the first cell cycle by mechanisms distinct from those used to establish them. PKC-3 phosphorylates PAR-2 and likely LGL-1 to prevent them from localizing to the anterior cortex [6], [7], [25] and PAR-2 prevents the accumulation of anterior PAR proteins in the posterior by an unknown mechanism possibly involving PAR-1 and PAR-5 [18], [25], [26]. Similarly, LGL-1 contributes to exclusion of the anterior PARs, but the mechanism is unknown [6], [7].

*C. elegans* regulates protein turnover through conserved ubiquitin-mediated degradation mechanisms [27]. Ubiquitin conjugation requires the activity of E1, E2, and E3 enzymes, and results in the covalent attachment of mono-ubiquitin or poly-ubiquitin to a target protein [28]. Modification by poly-ubiquitin often leads to protein degradation. Mono-ubiquitylation tends to regulate protein activity or acts as a signal for packaging into multivesicular endosomes [29], [30]. As with other post-translational protein modifications, ubiquitylation is reversible. Several classes of proteins have the ability to remove ubiquitin from specific substrates after it has been added, or to break up and recycle poly-ubiquitin chains [28], [30]. These enzymes are collectively called deubiquitylating enzymes, or DUBs. Deubiquitylation activity presents biochemical regulatory networks with the opportunity to temporarily modify proteins by removal of ubiquitin to recycle proteins otherwise destined for degradation, or to modulate their function. In this study, we explored the role of three putative deubiquitylating enzymes (DUBs) in embryo polarization. These three conserved DUBs, MATH-33, USP-46, and USP-47, function redundantly at the genetic level with regard to their functions in polarization of *C. elegans* one-cell embryos. Based on our analysis we propose that these DUBs function during polarity establishment by contributing to positioning the centrosome, and thereby promoting the cytoskeletal changes that initiate polarity establishment.

## Results

### Loss of function of *math-33* enhances weak *par* mutants

*math-33* was identified in an RNA interference (RNAi)-based screen for enhancers of embryonic lethality of weak *par-1* and *par-4* mutants [31]. *math-33* encodes a protein with a meprin and TRAF homology (MATH) domain, and a ubiquitin carboxy-terminal hydrolase (UCH) domain. Math-33 depletion increases the lethality of *par-1(zu310ts)*, *par-4(it57ts)*, and strongly increases lethality in three weak *par-2* mutant alleles, but not *lgl-1(tm2616)* or a partially suppressed *par-3(e2074)* nonsense mutation (Table 1). To determine whether enhancement of *par-1*, *par-2* and *par-4* conditional mutants simply reflected a general requirement for MATH-33 in embryos compromised by any conditional mutation, we tested eight other genotypes: *emb-9(g23ts), emb-9(b189ts), zyg-9(b288ts); unc-4(e120), mom-2(ne874ts); unc-5(e53), mom-4(ne1539ts), wrm-1(ne1982ts), mig-5(rh147ts), lit-1(ne1991ts)* (Table S1) [32]–[36]. We found that MATH-33 depletion could only enhance two of the tested mutations, indicating that *math-33* is not a non-specific enhancer. Two strains, *emb-9(g23ts) and zyg-9(b288ts);unc-4(e120)* showed significantly increased embryonic lethality. However a different allele of *emb-9, b189ts*, failed to show significant changes in embryonic lethality, indicating that different mutations or genetic backgrounds are more sensitive to comprising deubiquitylation. Because all three alleles of *par-2(it5ts, it87* and *e2030)* are enhanced strongly by depletion of MATH-33, we investigated the role of MATH-33 in polarity.

**Table 1:** Embryonic lethality of *par* mutants depleted of MATH-33 by RNAi.

Genotype	control*(RNAi)*		*math-33 (RNAi)*	
	Embryo lethality	*n*	Embryo lethality	*n*
N2 – wild type	1%	1606	4%	1428
*par-1(zu310ts)*	10%	303	35%*	657
*par-2(it5ts)*	6%	1433	97%*	1532
*par-2(it87) unc-32(e189)*	8%	211	78%*	422
*par-2(e2030) unc-32(e189)*	0%	179	74%*	412
*par-2(lw32) unc-45(e286ts)/ScI dpy-01 let-III*	1%	141	1%	120
*par-3(e2074); sup-7(st5)*	31%	886	32%	590
*par-4(it57ts)*	25%	590	60%*	541
*lgl-1(tm2616)*	0%	301	2%	359

*n* = the number of embryos counted for each genotype.

* *math-33(RNAi)* lethality different from control lethality by Student's t-test, p<0.05.

To determine whether MATH-33 has a specific role in polarity we examined the phenotypes of homozygous *math-33* mutants and asked whether the increased lethality of *math-33* when combined with *par-1, par-2* and *par-4* correlated with polarity defects. We obtained the probable null allele *math-33(tm3561)*, and found that homozygotes for the outcrossed mutation showed maternal effect, cold-sensitive and weakly penetrant embryonic lethality, along with weakly penetrant larval lethal and sterile phenotypes (Table 1, Figure S1). We found that RNAi of *math-33* in *par-2(it5ts)* at permissive temperature resulted in polarity phenotypes at frequencies typical of strong *par-2* mutants (Table 2), and that depletion of PAR-1 and PAR-4 by RNAi in *math-33(tm3561)* did not simply enhance the *par-1* and *par-4* polarity phenotypes but instead resulted in synthetic phenotypes that resembled *par-2* mutants (Table 2). For example, whereas neither *math-33(tm3561)* nor *par-1(RNAi)* alone exhibited the characteristic *par-2* phenotype of transverse spindle orientation in P1, in combination they resulted in 71% transverse P1 spindles. Together these data indicate that MATH-33 has a role in polarity and argue that the effect is most closely related to the function of PAR proteins that act in the posterior.

**Table 2:** Polarity phenotypes of *math-33; par* loss-of-function embryos.

Genotype	RNAi	*n*	AB-P1 Synchrony^a^	AB-P1 Spindle Orientations				AB area^c^
				T-L^b^	T-T^b^	L-T^b^	L-L^b^	Total
*N2*	*control*	25	0%	100%	0%	0%	0%	57%
*N2*	*par-1*	14	100%	100%	0%	0%	0%	54%
*N2*	*par-4*	14	21%	100%	0%	0%	0%	55%
*math-33(tm3561)*	*control*	23	13%	100%	0%	0%	0%	57%
*math-33(tm3561)*	*par-1*	24	100%	29%	71%	0%	0%	53%
*math-33(tm3561)*	*par-4*	34	47%	88%	12%	0%	0%	53%
*math-33(tm3561)*	*usp-47*	12	83%	42%	50%	0%	8%	50%
*par-2(it5ts)*	*control*	17	12%	76%	24%	0%	0%	58%
*par-2(it5ts)*	*math-33*	26	96%	4%	96%	0%	0%	54%
*par-3(e2074) lon-1; sup-7*	*math-33*	16	25%	69%	31%	0%	0%	55%
*par-3(e2074) lon-1; sup-7*	*control*	17	6%	35%	29%	29%	6%	54%

a proportion of embryos in which AB and P1 divided within 10 seconds of one another.

b Anaphase spindle orientations in two-cell embryos. T-L is the wild-type pattern with AB oriented transversely (T) and P1 oriented longitudinally (L).

c ratio of the area of an optical cross-section of the AB cell at mid-focal plane to the total embryo area in the same section (see materials and methods).

### The two DUBs MATH-33 and USP-47 are required for asymmetry in the one-cell embryo

**Figure 1:**
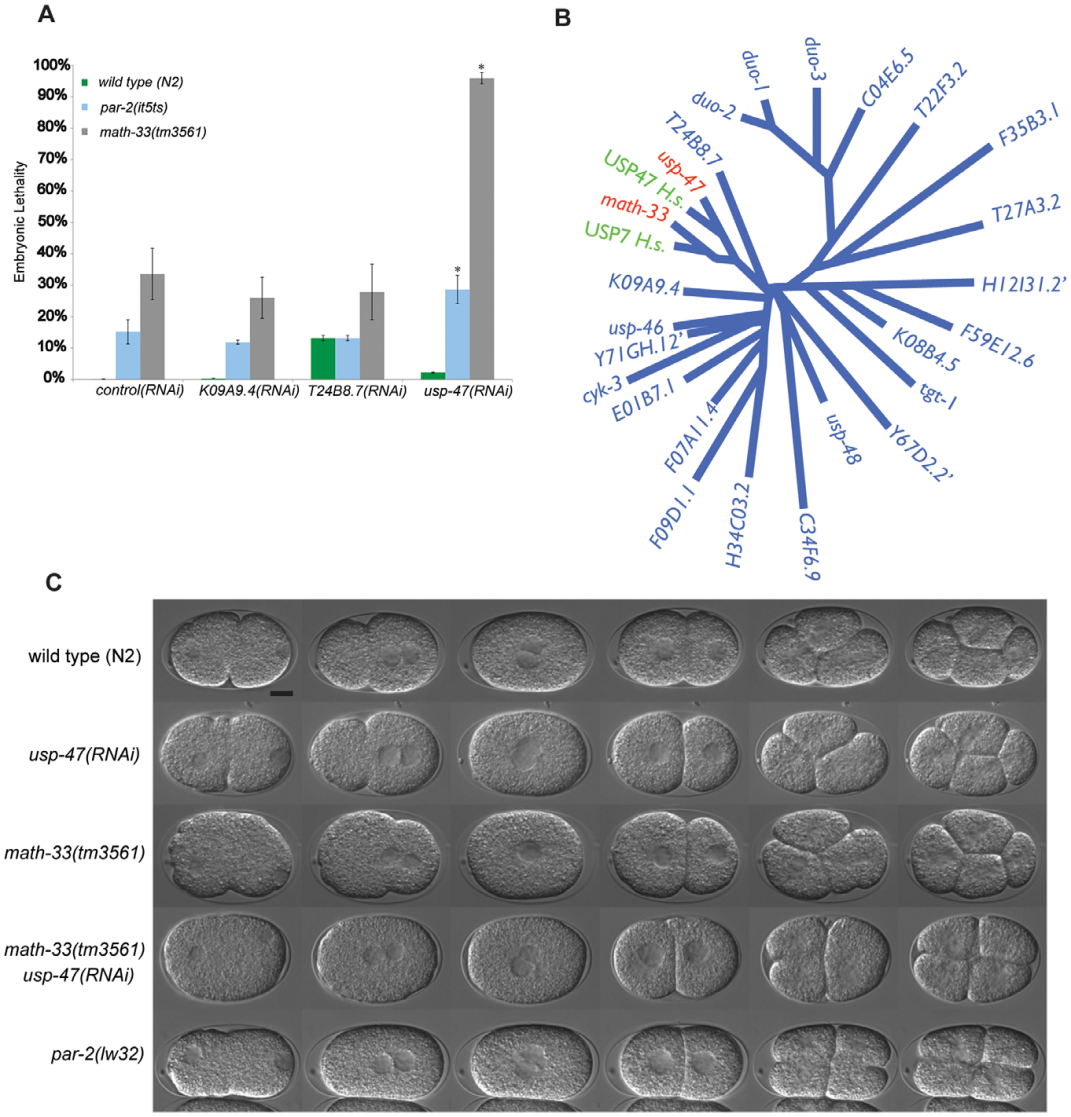
Paralogs *math-33* and *usp-47* have synthetic polarity phenotypes. (A) Bars indicate percent embryo lethality. >300 embryos were scored for each genotype in two trials. Error bars indicate standard error of the mean; asterisks indicate significant difference compared to controls, *p*<0.01, determined by Student's t-test. *K09A9.4* and *T24B8.7* are negative control DUBs. (B) Un-rooted phylogenetic tree of 25 UCH domains in *C. elegans* and two human homologs of *math-33* and *usp-47*. We did not perform RNAi on the three genes indicated by ‘. Human homologs USP7 and USP47 are included to highlight that there is evolutionary conservation of the DUBs between species. (C) DIC time-lapse of one-cell to four-cell embryos of the indicated genotypes at 22°C. Embryos are oriented with the anterior at the left in this and subsequent figures. The spindle orientation of P1 as it divides is usually longitudinal to the embryo axis, but is transverse in polarity-defective mutants such as *par-2(lw32)*. The scale bar represents 10 µm.

To address whether the genetic interaction between *par-2(it5ts)* and *math-33(RNAi)* is specific or whether other ubiquitin hydrolases also interact with *par-2*, we used RNAi to deplete 22 of 25 genes containing a Ubiquitin Carboxy-terminal Hydrolase (UCH) domain defining the class of DUBs to which MATH-33 belongs. We found that T05H10.1 RNAi depletion causes significant increases of *par-2(it5ts)* embryonic lethality (15% to 28%; Figure 1A). Furthermore, depletion of these 22 DUBs in *math-33(tm3561)* mutants revealed that depletion of T05H10.1, hereafter referred to as USP-47, caused synthetic lethality in the *math-33(tm3561)* mutant by increasing embryonic lethality from 35% to 95% (Figure 1A), and by increasing the lethality of a second probable null *math-33* allele, *ok2974*, from 45% to 93%. USP-47 is closely related to MATH-33 (Figure 1B) and appears to be a paralog of MATH-33 that is conserved among bilatera (TreeFam), whereas MATH-33 is a more widely conserved protein found in most eukaryotes. This conservation led us to hypothesize that these two genes have overlapping functions in embryonic polarity, which we tested by examining the phenotype of *math-33(-); usp-47(RNAi)* early embryos. We found that *math-33(tm3561); usp-47(RNAi)* and *math-33(ok2974); usp-47(RNAi)* two-cell embryos displayed variably penetrant polarity phenotypes similar to those of *par-2* mutants, including synchronous cell divisions, symmetry of cell size, and transverse spindles in both AB and P1, and occasional P1 cytokinesis defects (Table 2 and Figure 1C). Because *math-33(ok2974)* was indistinguishable from *math-33(tm3561)* in these assays, for simplicity we limited subsequent analysis to the *tm3561* allele.

**Figure 2:**
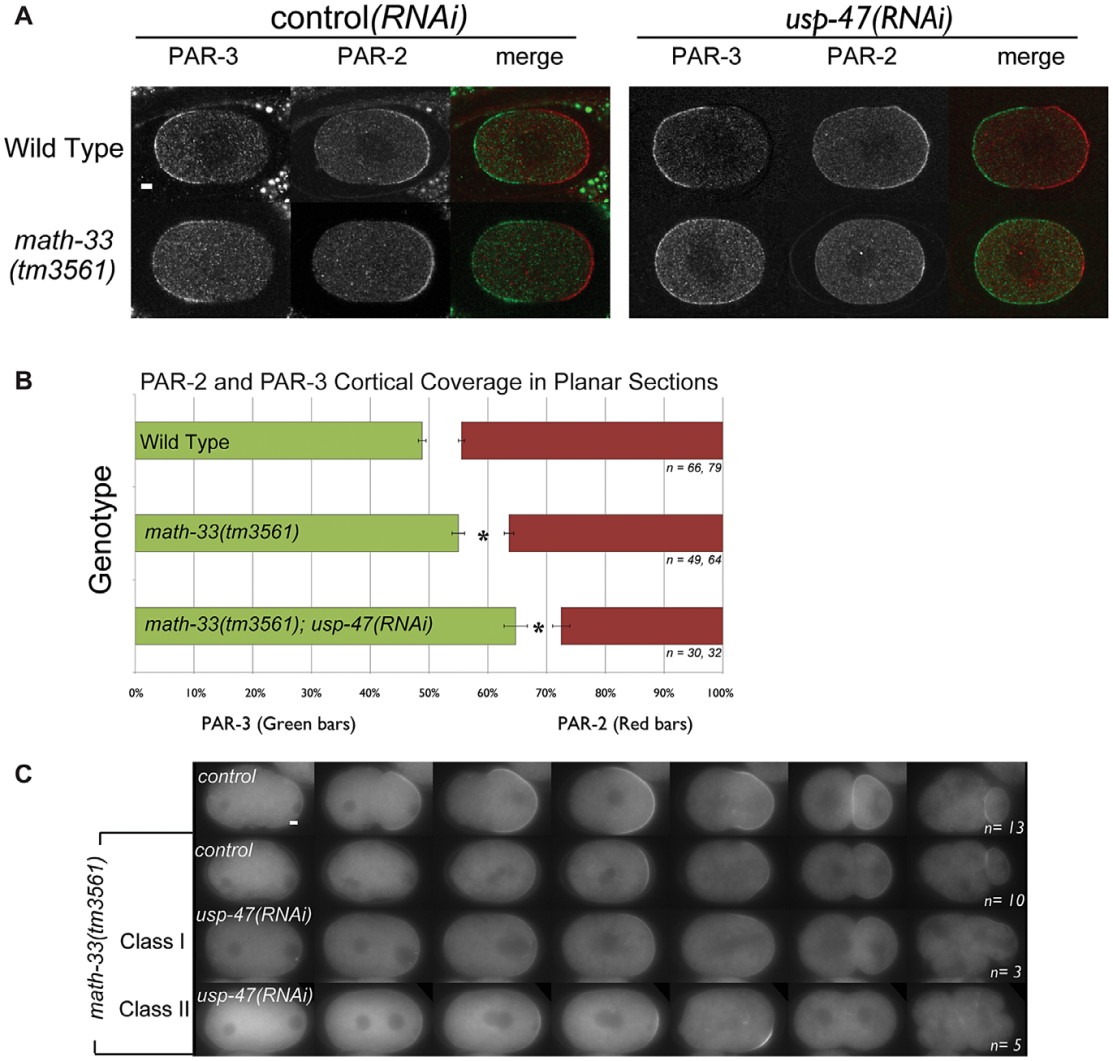
Polarity defects in *math-33(tm3561);usp-47(RNAi)* embryos. (A) Confocal micrographs of embryos immunostained for PAR-3 (green) and PAR-2 (red). Compared to controls, PAR-3 occupies a larger region of the cortex and PAR-2 a smaller region in the *math-33(tm3561); usp-47(RNAi)* embryo. (B) Averages of PAR-3 and PAR-2 domain sizes in immunostained embryos as a percentage of embryo length. *n* indicates the number of anterior and posterior domains examined respectively. Asterisks mark statistical significance compared to wild type, *p*<0.01, by Student's t-test. (C) Time-lapse images displaying the dynamic localization of PAR-2::GFP in the indicated genotypes. The embryo genotype of the control row (top) showing wild-type behavior is *par-2::gfp; math-33(tm3561)/nT1*. *math-33(tm3561); usp-47(RNAi)* embryos weakly recruit PAR-2 and could be grouped into two classes: those that maintain a PAR-2 domain throughout the cell cycle (class I), and those in which the weak PAR-2 domain is not maintained (class II). Scale bars represent 5 µm.

We hypothesized that the simultaneous loss of MATH-33 and USP-47 would affect the distribution of PAR-2 and PAR-3 at the cell cortex. One-cell embryos immunostained for PAR-2 and PAR-3 and scored between onset of pronuclear migration and centration indicate that *math-33(tm3561)* and *math-33(tm3561); usp-47(RNAi)* embryos exhibit a variable decrease in the size of the posterior cortical domain, and a reciprocal increase in the size of the anterior cortical domain (Figure 2A, 2B). This result illustrates that the loss of MATH-33 causes a very mild defect in PAR protein distributions at the one-cell stage and that the loss of both DUBs causes stronger defects. To determine the basis for the smaller posterior domain marked by PAR-2, we examined *math-33(tm3561); par-2::gfp; usp-47(RNAi)* embryos by time-lapse video microscopy, and observed two phenotypic classes (Figure 2C). In class I, PAR-2::GFP was recruited weakly to the cortex in a domain whose size was comparable to wild type and was maintained through the first cleavage. These embryos went on to divide normally. In class II, PAR-2::GFP was initially recruited to a smaller domain, which failed to expand and did not persist through cell division. These class II embryos exhibited polarity defects at first and second cleavages (Figure 2C). To determine whether there was a reciprocal effect on the anterior PAR domain, we attempted to construct a *math-33(tm3561)* strain expressing PAR-6::mCherry. Unfortunately, perhaps due to the over-expression of PAR-6, we were unable to maintain *math-33(tm3561); par-6::mCherry* worms in stock. However, *lgl-1::GFP; par-6::mCherry; math-33(tm3561)* worms are relatively healthy, perhaps because LGL-1::GFP expression counteracts the PAR-6::mCherry. Depletion of USP-47 in this strain resulted in a clearing of PAR-6::mCherry during establishment that was less robust than in wild type (Figure S2). The result that both PAR-6 clearing and PAR-2 localization in the posterior are impaired suggests that the establishment of a posterior domain is compromised in the absence of MATH-33 and USP-47.

### MATH-33 and USP-47 control polarity independently of PAR-2

**Figure 3:**
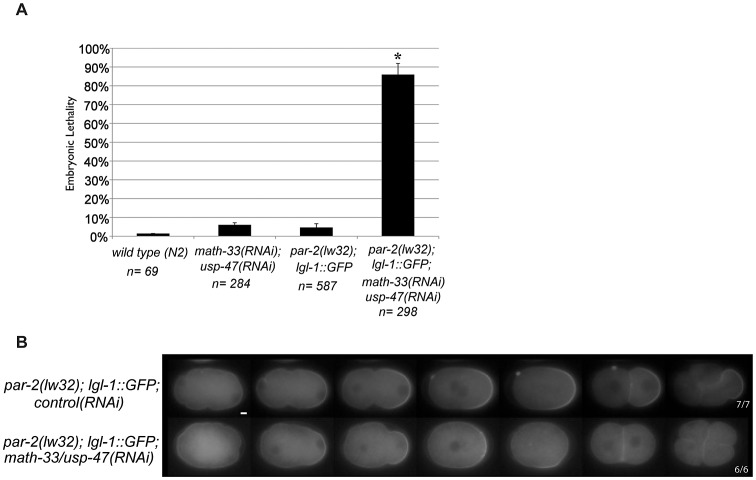
PAR-2 function is not required to mediate loss of polarity in *math-33(RNAi) usp-47(RNAi)*. (A) Embryonic lethality of the indicated genotypes. Error bars indicate the standard error of the mean. The experimental class marked with an asterisk is significantly different from all three controls, *p*<0.05, by Student's t-test. (B) LGL-1::GFP time-lapse images of embryos of the indicated genotypes. Upon knockdown of the DUBs MATH-33 and USP-47, the LGL-1::GFP domain size is smaller than in control embryos and is not maintained, similar to previous results with PAR-2::GFP in Figure 2C, and transverse spindles occur in P1. Scale bar represents 5 µm.

The sequence similarities between MATH-33 and USP-47 (Figure 1B) and their shared role in polarity raise the possibility that these two proteins act to deubiquitylate common substrates. PAR-2 is a putative E3 ubiquitin ligase that may self-regulate through auto-ubiquitylation [25]. Since *math-33(tm3561); usp-47(RNAi)* embryos resemble *par-2* mutants, we hypothesized that ubiquitylated-PAR-2 could serve as a regulatory target of the DUBs. To test this, we attempted to examine PAR-2 protein levels in embryo protein extracts, but were not able to obtain interpretable results. Instead, we tested whether the effects of the DUBs would be suppressed in a genetic background that bypassed the requirement for the PAR-2 protein: *par-2(lw32); lgl-1::gfp*. The *lw32* allele is a likely null allele; it contains a nonsense mutation that truncates the PAR-2 protein at amino acid 233 [37] resulting in absence of a domain required for cortical localization [25] and RNAi treatment of this allele does not increase the severity of the phenotype [6]. Overexpression of LGL-1 can bypass the absence of functional PAR-2 [6], [7]. We reasoned that if the DUBs acted by stabilizing PAR-2 the requirement for the DUBs would be suppressed by overexpression of LGL-1. We depleted MATH-33 and USP-47 simultaneously in the *par-2(lw32); lgl-1::gfp* strain and observed 86% embryonic lethality compared to 4% lethality in *par-2(lw32); lgl-1::gfp* control RNAi (Figure 3A). Embryos displayed a smaller posterior domain as revealed by LGL-1::GFP distribution, failed to maintain the posterior domain, and had transverse mitotic spindles in 6/6 P1 blastomeres (Figure 3B). We interpret these results to mean that the two DUBs do not act exclusively through PAR-2 to control polarity, and that their effects on the establishment of a posterior domain are likely to be parallel to the requirement for PAR-2.

### MATH-33 and USP-47 are required for myosin clearing in the one-cell embryo

**Figure 4:**
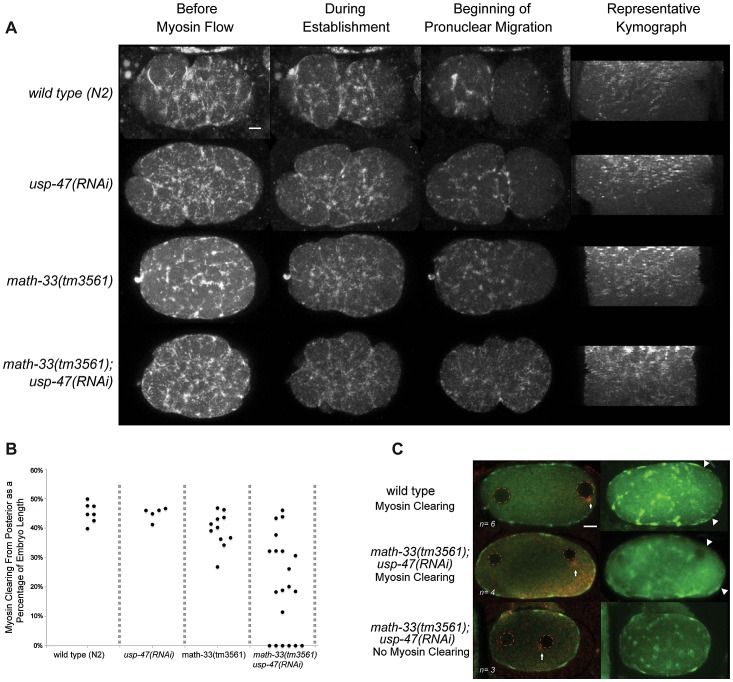
The posterior to anterior myosin clearing is defective in *math-33(tm3561); usp-47(RNAi)*. (A) Time-lapse confocal images of embryos expressing NMY-2::GFP. The maximum projections of sections through the cortex of the embryo show the localization of NMY-2::GFP foci at three stages. Kymographs illustrate the pattern of myosin clearing and flow over time. About halfway through the kymographs (from top to bottom), large myosin foci transition into smaller myosin puncta. (B) The extent of myosin absence from the posterior (clearing) was measured as a proportion of the embryo length and the maximum level of clearing was recorded; each data point represents one embryo. (C) Confocal stacks of embryos immunostained for NMY-2 (green) and tubulin (red). Arrows indicate the location of the centrosome, red dotted circles mark the position of pronuclei, and the extent of myosin clearing is indicated by arrowheads. Scale bars represent 5 µm.

Careful examination of *math-33(tm3561); usp-47(RNAi)* embryos revealed that in addition to *par-2-*like phenotypes, cortical contractility is decreased, embryos frequently lack pseudocleavage (Figure 1C), exhibit low penetrance cytokinesis defects (fewer than 2 in 10 embryos in most experiments), and occasional polar body extrusion defects (not quantified). The lack of pseudocleavage in this subset of embryos led us to hypothesize that absence of the DUBs may lead to defects in actomyosin function, which could lead to the failure to establish a posterior domain. To test this hypothesis, we observed cortical NMY-2 (type II non-muscle myosin heavy chain) [38] in 20 *math-33(tm3561); nmy-2::gfp; usp-47(RNAi)* embryos by confocal live imaging (Figure 4A, Videos S1, S2) and found that contractile myosin foci are present, but that the extent of myosin clearing, which we defined as the absence of foci from the posterior as a portion of the embryo length, was variably defective. Embryos showed a range in the extent of myosin clearing; 4/20 embryos showed normal clearing, 6/20 embryos no clearing and 10/20 embryos intermediate levels of clearing (Figure 4B). We confirmed this result by observing endogenous NMY-2 by immunostaining in embryos after pronuclear condensation but before pronuclear meeting, a stage when myosin clearing is readily detectable in wild type [14]. Three out of seven *math-33(tm3561); usp-47(RNAi)* embryos at this stage showed no evident clearing of myosin foci compared to none of six controls (Figure 4C). Furthermore, when we examined the velocity of myosin foci movement from posterior to anterior we found that wild type foci moved at a rate of 2.0±0.6 µm/minute (*n* = 13 foci from 6 embryos), whereas foci in *math-33(tm3561); usp-47(RNAi)* embryos moved at a rate of 1.5±0.7 µm/minute (*n* = 23 foci from 11 embryos *p* = 0.016 in a Student's t-test). The wild type rate is slower than previously reported rates [12], perhaps due to the lower temperature at which our observations were made. We also compared flow rates from embryos that we judged to have no clearing to those embryos with clearing and saw no significant rate differences (1.3±0.05 µm/minute *n* = 8 foci from 3 embryos with no clearing and 1.6±0.06 µm/minute *n* = 15 foci from 8 embryos with myosin clearing). This is consistent with previous observations that clearing is a consequence of flow away from the posterior as well as prevention of formation of new posterior foci [12] and implies that the activity of the DUBs is required for both.

### MATH-33 and USP-47 promote association of the centrosome with the cortex and attachment to the male pronucleus

**Figure 5:**
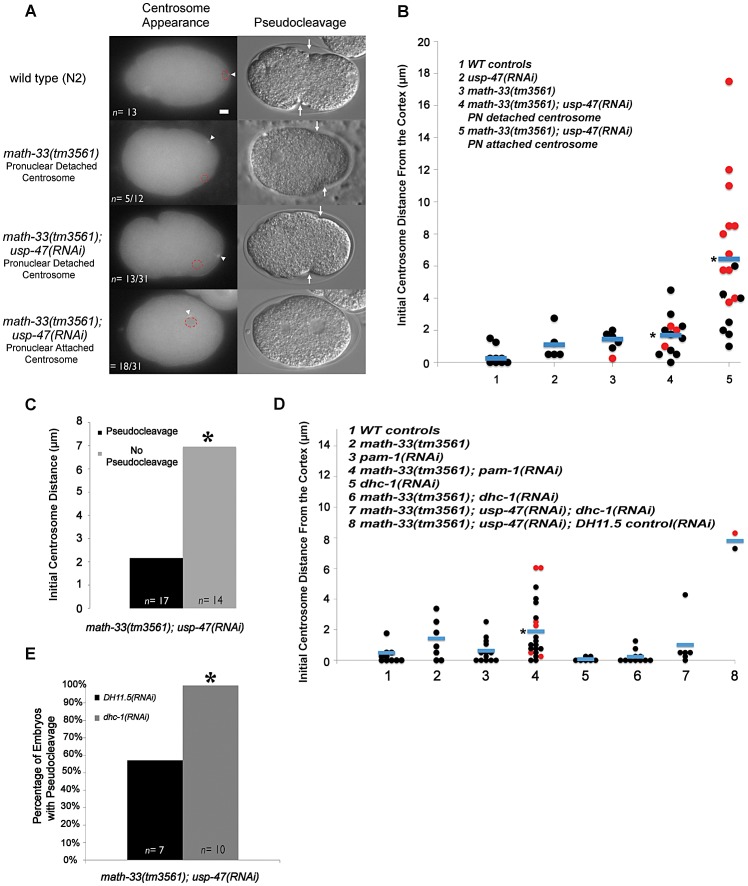
Deubiquitylases regulate the position of the centrosome in early one-cell embryos. (A) Micrographs showing tubulin::GFP in embryos at the time the centrosome was first detected. Centrosomes are marked with a white arrowhead and the position of the paternal pronucleus is indicated by red dotted circles. The DIC micrograph column shows representative embryos of the same genotypes at the time of pseudocleavage (an indirect indicator of the extent of myosin clearing). The white arrows indicate the location of pseudocleavage furrows. (B) Distance in micrometers of the centrosome from the embryo cortex at the time of earliest detection for the indicated genotypes. Each data point represents a single embryo. Black dots are embryos that had normal or weak pseudocleavage, red dots are embryos that displayed no pseudocleavage. Blue bars mark the mean distance. The asterisk indicates a significant difference from WT controls, *p*<0.01, by Student's t-test. (C) The number of embryos lacking pseudocleavage increases as centrosome distance from the cortex increases. Embryos were pooled from five RNAi experiment replicates. Differences were statistically significant according to a Student's t-test, *p*<0.01. (D) The distance of the centrosome from the cortex measured in the indicated genetic backgrounds. *p*<0.01 for column 4 compared to WT distances from multiple experiments, but *p* = 0.156 for column 4 compared to *math-33(tm3561)* in multiple experiments. (E) A single experiment examining absence of pseudocleavage resulting from depletion of DUBs. The absence of pseudocleavage phenotype is completely suppressed by *dhc-1(RNAi), p*<0.05. Scale bar represents 5 µm.

Because cortical flow is dependent upon a signal from the centrosome [12], we examined the position of the centrosome in fixed embryos immunostained for NMY-2 (Figure 4C). In this small data set we noted that the centrosome appeared to be significantly more distant from the cortex in *math-33(tm3561); usp-47(RNAi)* embryos (Figure S3A) and that this correlated with the extent of myosin clearing. However, because fixed embryos do not provide sufficient temporal resolution, we examined centrosome behavior in live embryos using beta tubulin::GFP to follow centrosomes. We assayed initial position of the centrosome relative to the male pronucleus and the cortex, and determined the time that the centrosome spent in close proximity to the cortex. In *math-33(tm3561); tbb-2::gfp; usp-47(RNAi)* embryos the position of the centrosome with respect to both the pronucleus and the cortex was variably abnormal (Figure 5A; Videos S3, S4, S5).

We observed two phenotypic classes with respect to pronuclear attachment. In some embryos, at first detection, the centrosome was attached to the male pronucleus (*n* = 18/31 from multiple experiments, Figure 5B, Video S4). In the other embryos the centrosome was detached from the pronucleus (*n* = 13/31 from multiple experiments, Figure 5B, Video S5). In all 13 cases in which centrosomes were initially detached from the male pronucleus, the centrosome and pronucleus eventually migrated toward one another and re-associated (Video S5). The centrosome was also detached from the pronucleus in 5/14 *math-33(tm3561); tbb-2::gfp* embryos. There is no statistical difference in the number of detached centrosomes between *math-33(tm3561)* and *math-33(tm3561); usp-47(RNAi)*. Because the frequency and severity of polarity defects is much lower in *math-33(tm3561)* embryos, this argues that centrosome attachment to the male pronucleus is not essential for polarity establishment.

Measurements of centrosome-to-cortex distances in *wild-type*, *math-33(tm3561)* and *math-33(tm3561); usp-47(RNAi)* are presented in Figure 5B. In wild type embryos the centrosome at first appearance is within 2 µm of the cortex with an average distance of 0.25 µm (*n* = 9). In *math-33(tm3561)* and in *usp-47(RNAi)* the average distance from the cortex (1.4 µm *n* = 5 and 1.1 µm *n* = 11 respectively). *math-33(tm3561)* centrosomes are significantly more distant than *wild type*, but have an average centrosome distance that is within *wild type* range. In contrast, centrosomes in *math-33(tm3561);usp-47(RNAi)* showed a wide range of distances. When we separated the two classes of embryos with respect to centrosome attachment to the pronucleus (last two columns in Figure 5B) we noted that when the centrosome was attached to the male pronucleus, it was more likely to be far from the cortex. Indeed, the average centrosome-cortical distance of 1.7 µm (*n* = 13) in those *math-33(tm3561); usp-47(RNAi)* embryos with detached centrosomes, and in those embryos with attached centrosomes, the average distance was significantly further, 6.4 µm (*n* = 18, *p*<0.001).

To determine whether the initial distance of the centrosome from the cortex correlated with polarity defects, we examined pseudocleavage, an indirect indicator of polarity establishment, in these same embryos. We defined absence of pseudocleavage as the absence of detectable persistent medial cortical invaginations in mitotic prophase. Embryos lacking pseudocleavage are indicated by red dots in Figure 5B. In the 17 *math-33(tm3561);usp-47(RNAi)* embryos in which pseudocleavage was detectable, the distance of the centrosome from the cortex was on average 2.1 µm, whereas in the 14 embryos lacking detectable pseudocleavage, the average distance was significantly larger, 7.0 µm (Figure 5C).

We saw no correlation between detached centrosomes and defects in polarity as assayed by absence of pseudocleavage. Most detached centrosomes from *math-33(tm3561); usp-47(RNAi)* embryos first become visible within 4 µm of the cortex, and amongst these; 25% (*n* = 12) failed to undergo pseudocleavage, For embryos with attached centrosomes within 4 µm, 28% (*n* = 7) embryos failed to undergo pseudocleavage. Therefore, the detachment status of the centrosome does not appear to influence whether polarity establishment will occur.

Because the centrosome's dwell time at the cortex can also affect polarity [23], [24], we compared the time that centrosomes spend in close proximity to the cortex in *wild type*, *math-33(tm3561)* and *math-33(tm3561);usp-47(RNAi)* embryos. In the wild type embryos centrosomes stayed within 4 µm of the cortex for an average of 8.8 minutes (*n* = 3). In contrast, *math-33(tm3561); usp-47(RNAi)* embryos exhibited dwell times of 2.5 minutes (*n* = 6) and *math-33(tm3561)* embryos spent an average of 5.8 minutes (*n* = 3). Thus, dwell time at the cortex is also shortened by loss of deubiquitylation activity.

In summary, we find that *math-33(tm3561); usp-47(RNAi)* embryos have abnormal centrosome behavior patterns that are consistent with the centrosome having a reduced affinity for the cortex and for the pronucleus. Because distance from the cortex correlated with failure of pseudocleavage and previous work showed that dwell time at the cortex correlated with polarity defects, we hypothesized that defects in centrosome-cortex interaction are responsible for the observed defects in polarity establishment.

### Depletion of dynein heavy chain bypasses the requirement for MATH-33 and USP-47

To test the hypothesis that weakened centrosome-cortex interaction caused the polarity defects in *math-33(tm3561); usp-47(RNAi)* we used *dhc-1(RNAi)* to deplete dynein, which causes centrosomes to strongly localize at the cortex [24], and scored polarity phenotypes and centrosome behavior. We reasoned that if the defect in cortical association was causing the polarity defect, then forcing a tight association of the centrosome and cortex would suppress the polarity defects. Depletion of DHC-1 in both *math-33(tm3561)* and *math-33(tm3561); usp-47(RNAi)* resulted in a closer initial association of the centrosome to the cortex (Figure 5D). We also found that 10/10 *math-33(tm3561); usp-47(RNAi); dhc-1(RNAi)* embryos displayed robust pseudocleavage, whereas only 4/7 *math-33(tm3561)* embryos simultaneously treated with *usp-47(RNAi)* and *DH11.5(RNAi)*, a control for the non-specific effects of double RNAi, had pseudocleavage (Figure 5E). Thus, influencing the centrosome position to bring it closer to the cortex suppresses *math-33; usp-47(RNAi)* polarity phenotypes, consistent with the hypothesis that the primary effect of the DUBs on polarity is to promote association of the centrosome with the cortex.

### PAM-1 depletion enhances the phenotype of *math-33*

The gene *pam-1* is known to affect centrosome dynamics in the early embryo [23], [24] in a way that is similar, but not identical to, the loss of the DUBs. Loss of PAM-1 does not appear to affect the initial proximity of the centrosome to the cortex, but like the loss of the DUBs, results in premature departure of the centrosome from the cortex [23]. To test for possible genetic interaction between *pam-1* and *math-33*, we examined centrosome behavior and pseudocleavage in *math-33(tm3561); pam-1(RNAi)* embryos expressing tubulin::GFP. We found that *pam-1(RNAi)* enhanced centrosome defects in *math-33* in a way similar to *usp-47(RNAi)*. Relative to *math-33(tm3561)* or *pam-1(RNAi)* alone, we noted an increase in the fraction of centrosomes that were located further from the cortex (Figure 5D), an increase in the frequency of centrosomes detached from the nucleus (Figure S3B), as well as more frequent lack of pseudocleavage (red dots in Figure 5D). As a control we also depleted PAR-2, a protein not known to affect the centrosome position, in *math-33(tm3561)*. PAR-2 depletion had no effect on the initial distance of the centrosome from the cortex, and did not cause any synthetic effects that led to the loss of pseudocleavage (Figure S3C). Thus loss of *pam-1* enhances *math-33* centrosome and polarity phenotypes.

### MATH-33 and USP-47 are expressed in early embryos and the germline

**Figure 6:**
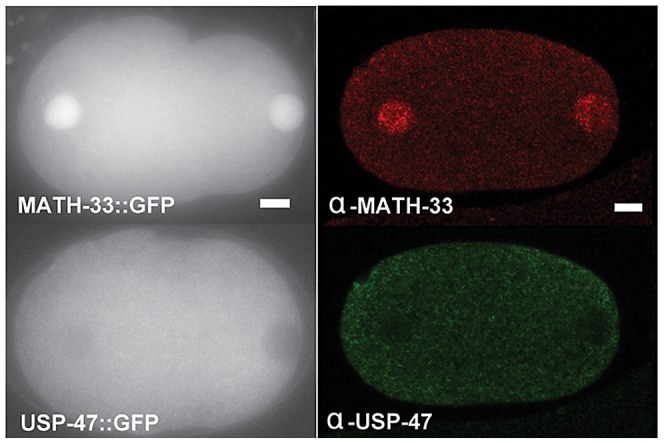
MATH-33 and USP-47 are not enriched at cortex or centrosome. The left panels are representative images of embryos showing the distribution of GFP::MATH-33 and GFP::USP-47 in one-cell embryos. Both proteins are present in the cytoplasm, but MATH-33 is enriched in nuclei. The right panels are images of embryos showing immunostaining of endogenous MATH-33(red) in the cytoplasm and the nucleus; USP-47(green) primarily in the cytoplasm. Scale bar represents 5 µm.

In an effort to gain insight into possible targets of MATH-33 and USP-47, we examined expression patterns and localization of MATH-33 and USP-47 in embryos and adult worms. We found that anti-MATH-33 and anti-USP-47 antibodies and *pie-1* promoter driven MATH-33::GFP and USP-47::GFP transgenic worm strains gave consistent results in early embryos: MATH-33 is present at high levels in both cytoplasm and nucleus, whereas USP-47 is only detected at high levels in the cytoplasm (Figure 6) indicating that the cytoplasm is the most likely site of action of the two DUBs. Furthermore, antibody staining showed that MATH-33 is present in most or all cells in the worm (not shown) and is enriched in the germline (Figure S4A), whereas USP-47 is present primarily in the germline (Figure S4B). Expression of *Ppie-1::math-33::gfp* in the germline of *math-33(tm3561)* mutants was able to suppress lethality and sterility phenotypes (Figure S1B), indicating that the maternal contribution of MATH-33 to embryos is sufficient to compensate for most essential MATH-33 functions.

### MATH-33 and USP-47 antagonize protein degradation

**Figure 7:**
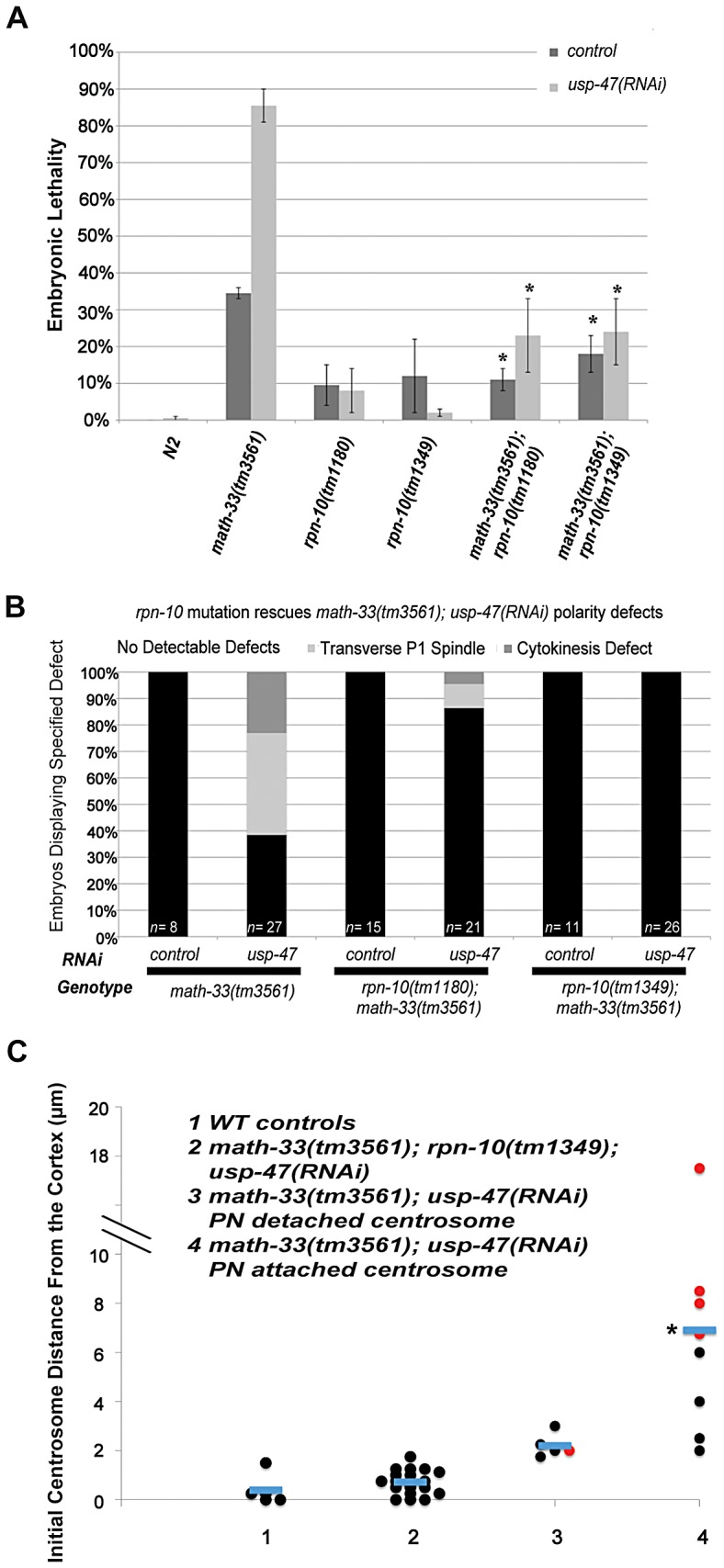
Mutation of *rpn-10* suppresses lethality and polarity defects in *math-33(tm3561); usp-47(RNAi)* embryos. (A) Embryonic lethality of *math-33(tm3561); usp-47(RNAi)* is reduced by either of two *rpn-10* mutations. Standard error of the mean is indicated by the error bars. *n*>350 embryos for N2 controls and *n*>600 for the other genotypes. Asterisks indicate significance compared to *math-33(tm3561)* single mutants, *p*<0.01, Student's t-test (B) Data showing the suppression of phenotypic defects in early *math-33(tm3561);usp-47(RNAi)* embryos by *rpn-10* mutations. (C) Distance in micrometers of the centrosome from the embryo cortex when it is first detectable. *rpn-10* mutation suppresses the absence-of-pseudocleavage phenotype and the mislocalization of the centrosome compared to *math-33(tm3561); usp-47(RNAi)* controls. None of the 18 centrosomes observed in column 2 were detached from the paternal pronucleus, indicating that the detachment phenotype was also completely suppressed. Results in column 4 were significantly different in a Student's t-test *p*<0.01 compared to column 1 controls. Data from columns 1, 3, and 4 are also displayed in Figure 5C.

The presence of a UCH domain in MATH-33 and USP-47 suggest that these proteins act as deubiquitylation enzymes. Removal of ubiquitin could have different consequences: a) prevention of degradation, b) modulation of protein activity c) effects on endocytosis or recycling of membrane proteins [29], [30]. We tested whether the major action of MATH-33 and USP-47 was to antagonize poly-ubiquitylation by asking whether compromising protein turnover could suppress the lethality and polarity defects of *math-33(tm3561); usp-47(RNAi)* worms. We compromised protein degradation by crossing *rpn-10(tm1349)* or *rpn-10(tm1180)* into *math-33(tm3561)* worms. RPN-10, like its baker's yeast homolog [39], [40], is a non-essential ubiquitin recognition protein; although its relationship to proteasome function is unclear, mutants in *rpn-10* result in abnormal accumulation of poly-ubiquitylated protein [41], [42]. Mutants in *rpn-10* were also found to be able to suppress phenotypes of *par-2(it5ts)* [42]. Our results show that *rpn-10* mutations strongly suppress *math-33(tm3561); usp-47(RNAi)* embryonic lethality and polarity phenotypes (Figure 7A, 7B). Because compromising protein degradation could affect polarity in a number of ways, we asked whether *rpn-10* mutations also suppressed the centrosome positioning defects in *math-33; usp-47(RNAi)* embryos. We found that centrosomes in *rpn-10(tm1349); math-33(tm3561); usp-47(RNAi)* embryos were indeed always found very close to the cortex, similar to wild type (Figure 7C, Video S6). Therefore, we conclude that in their role in early embryonic polarity, the DUBs are likely to regulate protein turnover.

### USP-46 also functions redundantly with MATH-33 and USP-47

**Figure 8:**
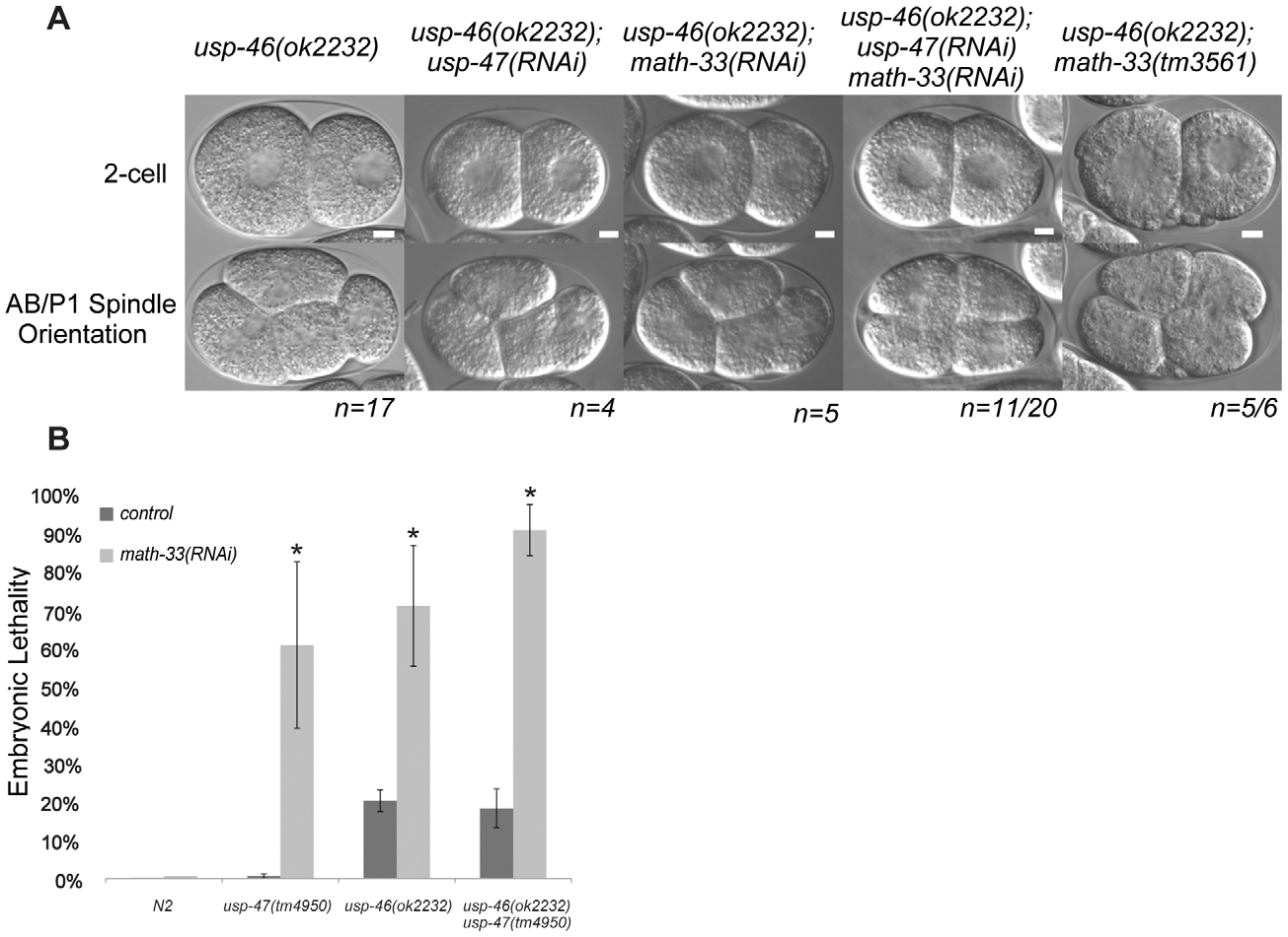
*usp-46* acts redundantly with *math-33* and *usp-47*. (A) Two-cell embryos at interphase (top) illustrate unequal vs. equal first divisions and at P1 mitosis (bottom) show spindle orientations. Maternal genotypes are indicated. Scale bars for each genotype represent 5 µm. (B) Embryonic lethality measured after depleting *math-33* in *usp-46* and *usp-47* mutants. Bars marked with an asterisk are significantly different from the RNAi controls in a t-test, *p*<0.01. Scale bar represents 5 µm.

A recent paper from Kouranti and colleagues [43] identified a novel and redundant role for a group of five DUBs, including homologs of *math-33* and *usp-47*, in polarity of *Schizosaccharomyces pombe*. Their results suggested to us that the role of this class of deubiquitylases in polarity could be evolutionarily conserved. The yeast proteins Ubp15p and Ubp5p are homologs of MATH-33 and USP-47 (Figure S5B). Because of the link between the yeast DUBs and polarity, we hypothesized that homologs of (*ubp4, ubp9*), the two other *S. pombe* UCH-containing DUBs that affect polarity might also function in *C. elegans* polarity. *C. elegans* USP-46 and E01B7.1 are homologs of the yeast proteins UBP9 and UBP4, respectively (Figure S5C). RNAi depletion of neither gene increases embryo lethality in wild type or in *math-33(tm3561)* (data not shown). However, a deletion mutant allele *usp-46(ok2232)* in combination with simultaneous *math-33(RNAi)* and *usp-47(RNAi)* resulted in 75% embryonic lethality (*n = 1330*), compared to 30% lethality (*n = 1408) in math-33(RNAi); usp-47(RNAi)* in wild type. Of 20 *usp-46(ok2232)* embryos in which MATH-33 and USP-47 were simultaneously depleted, 11 displayed transverse P1 spindle orientations (Figure 8A) compared to 1/30 *math-33(RNAi); usp-47(RNAi)* in wild type. However, *math-33(tm3561); usp-46(ok2232)* double mutants are largely sterile, producing only a few oocytes and no fertilized eggs, raising the possibility that the two genes act redundantly in gametogenesis. After mating to wild-type males, the *math-33(tm3561); usp-46(ok2232)* mutants can produce a few fertilized embryos which fail to hatch. Five of the six one-cell embryos we were able to obtain in this way displayed transverse P1 spindle orientations at the two-cell stage (Figure 8A) indicating defects in polarity. More recently a new deletion mutation, *usp-47(tm4950)*, became available; we found that double mutants of *usp-47(tm4950); usp-46(ok2232)* are unhealthy and display mild embryonic lethality and sterility. At 16°, these double mutants have no evident polarity defects, but at 25° some embryos display transverse spindles in P1 (4/18, Table 3), while the single mutants do not. *usp-46; usp-47* mutant embryos depleted for *math-33* display a high penetrance of transverse spindle orientations (11/15; Table 3), and embryos appear to be similar in most respects to *math-33(tm3561); usp-47(RNAi)* embryos (see Figure 1B). Taken together, the increasing penetrance of polarity phenotypes as a function of loss of activity of the three deubiquitylases (Table 3) suggests that the three enzymes function redundantly in early polarity, but that they vary in their contributions to the polarization process.

**Table 3:** Effect of RNAi/mutant combinations of *math-33*, *usp-46*, and *usp-47* on P1 spindle orientation.

Temperature	Genotype	T-T	*n*
16°	*N2*	0%	25
16°	*usp-46(ok2232)*	0%	25
16°	*usp-47(tm4950)*	0%	13
25°	*usp-46(ok2232)*	0%	9
25°	*usp-47(tm4950)*	0%	9
25°	*math-33(tm3561)*	0%	23
16°	*usp-46(ok2232); usp-47(tm4950)*	0%	23
16°	*N2; usp-47(RNAi) math-33(RNAi)*	3%	30
25°	*usp-46(ok2232); usp-47(tm4950)*	22%	18
16°	*math-33(tm3561); usp-47(RNAi)*	64%	28
16°	*usp-46(ok2232); usp-47(RNAi) math-33(RNAi)*	65%	20
16°	*usp-46(ok2232); usp-47(tm4950); math-33(RNAi)*	73%	15
16°	*usp-46(tm3561); math-33(tm3561)*	83%	6

T-T indicates that both AB and P1 cells of two-cell stage embryos divided transversely. The RNAi and mutant combinations of different DUB genes shown here are ordered according to the phenotypic penetrance of the transverse mitotic spindle orientations in P1.

## Discussion

Ubiquitin regulation appears to have an important but not well-understood role in *C. elegans* embryonic polarization. One of the earliest discoveries was that PAR-2 has homology to RING domain E3 ubiquitin ligases, suggesting that ubiquitin ligase activity may be important for excluding anterior PARs from the posterior [5], [37]. Hao and colleagues showed that the PAR-2 RING domain is required for robust transgene rescue of embryos lacking endogenous PAR-2, indicating that it is likely to be an active ubiquitin ligase *in vivo*, although this activity is not absolutely essential for function [25]. Biochemical targets of PAR-2, however, are unknown. Other results that relate ubiquitin-based regulation to polarization include the finding that PAR-6 levels are affected by activity of the ubiquitin ligase CUL-2 and its adapter protein FEM-3 [44], and that mutations of *C. elegans* homologs of the BRAT family of ubiquitin ligases have been shown to be able to suppress weak *par-2* phenotypes [45]. Weak impairment of protein turnover through mutation of *rpn-10* has also been shown to suppress *par-2* phenotypes [42], and depletion of the proteasome regulatory subunit, *rpn-2* results in abnormal spindle orientation in AB at the two-cell stage [46].

We report here additional evidence for an important role of ubiquitylation in embryonic polarity. We show that a group of three putative deubiquitylating enzymes, MATH-33, USP-46 and USP-47, contribute to polarity establishment in *C. elegans*, likely in a redundant fashion. Analysis of *math-33(tm3561); usp-47(RNAi)* embryos revealed that when the products of these two genes are missing or reduced, PAR protein domains are abnormally sized, cortical actomyosin flow is weak or fails, and centrosomes are variably positioned with respect to the cell cortex. Because distance of centrosomes from the cortex correlates with the severity of the polarity phenotypes, and because blocking movement of the centrosome away from the cortex by depleting dynein heavy chain restores polarity establishment in *math-33(tm3561); usp-47(RNAi)*, we propose that the primary role of the DUBs in polarity is to promote the association of the centrosome and the cortex.

Prior to polarization in the wild-type *C. elegans* embryo, the centrosome at first detection using the centrosome marker SPD-2 can be observed within a 0–9 *u*m range (5 *u*m average) from the cortex [11], [47]. It then moves to within 0–4 *u*m at the time of polarity initiation [11], [47]. In embryos lacking functional MATH-33 and USP-47 the position of the centrosome when first detected is often distant from the cortex and these unassociated centrosomes remain so. Furthermore, centrosomes in embryos lacking these two DUBS that are initially close to the cortex leave the cortex sooner than in wild type embryos. Therefore, we suggest that forces that maintain close association of the centrosome and cortex are weakened or absent and as a result, polarity establishment as assessed by myosin clearing and pseudocleavage is either weak or non-existent. However, we also see that centrosomes are often detached from the paternal pronucleus, indicating that the pronuclear-centrosomal interaction is defective. Centrosome-nuclear attachment is in many cases unnecessary for the proper localization of centrosomes in cells [48], and indeed we see that detached centrosomes can associate closely with the cortex and see no correlation between nuclear detachment and polarity defects. However, centrosomes that are detached from the nucleus are more likely to be closely apposed to the cortex and centrosomes that are attached to the paternal pronucleus are more likely to be more distant from the cortex. This correlation suggests that weakened centrosome interactions with cortex and pronucleus creates a competition between the two for binding to the centrosome. Because centrioles enter the embryo in association with the sperm pronucleus, detachment from the pronucleus must occur between the time that the embryo is fertilized and when TBB-2::GFP first allows the centrosome to become visible. Furthermore, all detached centrosomes are capable of re-attaching to the paternal pronucleus in a manner similar to maternal pronuclear capture by the growing astral microtubules. This suggests that a microtubule-mediated tracking mechanism [48] in which pronuclei migrate towards the centrosome on astral microtubules functions in a relatively normal way. Because of this, we speculate that the early centrosomal attachment to the pronucleus uses a different mechanism than the microtubule-based mechanism that promotes attachment during prophase. We also propose that the action of the tracking mechanism contributes to the early departure of the centrosomes from the cortex in the embryos lacking DUBs and having initially detached centrosomes.

We observed a few cases in which embryos with centrosomes closely apposed to the cortex had polarity defects. This could be due to shortened time of cortical association, or could indicate that factors other than centrosome-cortex association are affected by the loss of the DUB activity.

Our results are consistent with previous reports correlating centrosome proximity to the cortex with robust polarity establishment [23], [24] However, they are in apparent contradiction to a recent study concluding that centrosomes can initiate polarity establishment at a distance from the cortex [47]. This apparent contradiction can be reconciled by distinguishing between the initial signaling event, (referred to as “symmetry breaking” [41]) and an ongoing process of polarity establishment. Our results are consistent with a model in which the initial signaling event can happen at a distance from the cortex. Indeed we did not observe any *math-33; usp-47* double loss of function embryos in which a PAR-2 domain failed to be established. Although this could be explained by incomplete USP-47 RNAi knockdown, or by genetic redundancy in regulation of the targets of the DUBs, an equally likely interpretation is that the DUBs do not affect the early signaling event, but rather affect events downstream of that signal. The relationship we note between centrosome proximity and robust polarity establishment supports the notion that these downstream events involve continued interaction of the cortex with the centrosome or its associated astral microtubules or both and that this interaction is most efficient when the centrosome and cortex are more closely apposed.

The establishment phase of polarity is certainly affected by the loss of MATH-33 and USP-47. However we also observed distinct defects in polarity maintenance such as loss of PAR-2::GFP and LGL-1::GFP domains at the posterior cortex after prophase. The loss of maintenance is probably caused by re-entry of the anterior PARs into the posterior domain and could occur for two reasons. One is that *math-33(tm3561); usp-47(RNAi)* causes the initial clearing of myosin to be impaired, and thus the initial size of the posterior domain to be smaller. Afterwards, maintenance could fail due to the inability of the reduced amount of the posterior PARs to exclude anterior PARs. A second possibility is that the DUBs actively participate in maintaining the posterior domain. However there is less evidence of a role for MATH-33 in maintenance. LGL-1 has been proposed to primarily have an active role in maintenance only [6], and we found that although MATH-33 depletion increases polarity defects in *par-2(it5ts)* there are no effects on *lgl-1(tm2616)* mutants.

We observed variable penetrance and expressivity of the mutant phenotypes in the *math-33; usp-47(RNAi)* embryos. Three explanations for this variability are possible. First RNAi depletion of USP-47 could be incomplete. Second, the DUBs could play a modulatory role rather than an essential role, such that even a complete loss of function would not result in a fully penetrant phenotype. Third, the targets of the two DUBs are under redundant regulatory control; indeed our finding that USP-46 functions redundantly with MATH-33 and USP-47 supports this notion. We found that one of the strongest combinations of DUBs was the *usp-46; usp-47* double mutants depleted for MATH-33. Although a triple mutant combination could resolve the issue, obtaining triple mutants is not feasible because *math-33(tm3561); usp-46(ok2232)* worms are sterile.

Other proteins are also known to be required for proper interaction of the centrosome with the cortex in one-cell *C. elegans* embryos: dynein components and regulators, such as dynein heavy chain [49], and a puromycin sensitive aminopeptidase (PAM-1) [23], [24]. Reduction or loss of function of neither the dynein group nor PAM-1 precisely mimics the phenotypes resulting from the loss of DUBs. In embryos severely depleted of dynein heavy chain, 15% of centrosomes can become detached from the pronucleus, and in most embryos the centrosomes become tightly and persistently associated with the cortex [24]. Thus a key function of dynein is to positively promote dissociation of the centrosome from the cortex, but also to promote association with the pronucleus. In contrast MATH-33 and USP-47 promote cortical association and pronuclear attachment. In *pam-1* mutant embryos the centrosome-pronuclear complex is correctly positioned at the posterior cortex, but the complex spends less time at the cortex, leaving early, leading to polarity establishment defects [23], [24]. Because both the centrosome association and pronuclear attachment are mildly enhanced in *math-33(tm3561);pam-1(RNAi)* embryos it seems likely that the two proteins affect both processes; our experiments, however, do not allow us to distinguish whether the DUBs and PAM-1 act through a common pathway or affect centrosome dynamics through two separate pathways. A proposed function of PAM-1 is that it removes N terminal peptides from proteins to allow them to be ubiquitylated [23]. If this is the case, it is not clear how the DUBs would work with PAM-1 in a common pathway.

The DUBs appear to act by protecting proteins from degradation rather than by modifying protein activities or localizations. Evidence for this is that *rpn-10* mutations are effective suppressors of *math-33(tm3561); usp-47(RNAi)*. Mutations in *rpn-10* lead to an increase in the amounts of poly-ubiquitylated proteins and result in higher steady state levels of the protein TRA-2 [41]. By analogy with its homologs in other organisms, RPN-10 could act to recognize a subset of ubiquitylated substrates at the proteasome, and therefore mutating *rpn-10* may compromise efficacy of degradation of a subset of proteins at the proteasome [40], [50]. *rpn-10* mutation corrects the centrosome association with the cortex as well as attachment to the pronuclear envelope suggesting that it fully suppresses all centrosome position phenotypes associated with loss of the DUBs. In contrast, depletion of dynein heavy chain suppresses the defects in centrosome-cortex association and polarity, but does not suppress pronuclear envelope detachment. This indicates that phenotypic suppression by depleting dynein likely bypasses the normal regulatory target of the DUBs. A unifying hypothesis would be that the DUBs are required to maintain an appropriate level of one or more key proteins required for centrosomes to interact with the pronucleus and the cortex; alternatively the cortical association defects could be independent of the pronuclear attachment defects and the two be mediated by different proteins. There are many types of proteins that could be necessary for the centrosome position. The simplest hypothesis is that these proteins are centrosome components, but cytoskeletal motors, regulatory proteins such as kinases, and proteins that mediate interactions between microtubules and nuclei or cortical proteins are also candidates.

Comparison of our results with results from studies of homologous proteins in the fission yeast *Schizosaccharomyces pombe* raises the possibility that these DUBs have a conserved role in eukaryotic cell polarity. A screen in *S. pombe* of DUB function showed that *ubp4, ubp5, ubp9, ubp15*, and *sst2*, although non-essential individually, act redundantly to affect asymmetric endocytosis [43]. Because this group contained two proteins homologous to MATH-33 and USP-47, Ubp5p and Ubp15p, we speculated that if the functions were evolutionarily conserved, homologs of the other members of the group might have redundant roles in *C. elegans*, and led us to the discovery of a role for USP-46, the homolog of UBP9p, in *C. elegans* embryos. In *S. pombe*, microtubules are required to control proper polar growth [51], [52], so there may be an underlying common mechanism that involves the centrosome. Alternatively, since Kouranti *et al.* [43] noted strong phenotypes related to endocytosis, it may be possible that membrane proteins in *C. elegans* that contribute to cytoskeletal regulation are regulated by the DUBs.

The DUBs we examined appear to have both overlapping and distinct functions that may extend to diverse biological processes. *math-33(tm3561)* mutants display mild polarity phenotypes, whereas *usp-46(ok2232)* and *usp-47(RNAi)* do not. This indicates *math-33* individually plays a more crucial role in regulating *C. elegans* polarity than the other two DUBs. It is unlikely, however, that *math-33's* role is limited to early embryo polarity establishment. After nine outcrosses, *math-33(tm3561)* mutants remain pleiotropic, displaying embryonic lethality, larval lethality, and sterility. Furthermore, *math-33* has been reported to interact genetically with *vab-10, ksr-1*, and *skn-1* in *C. elegans* [53]–[55], and as we report, *emb-9*, and either *zyg-9* or *unc-4*. This indicates that *math-33* is likely to act in several biological pathways that may not relate directly to polarity. MATH-33 homologs in other organisms provide little insight on an exact mechanism of action in polarity, but suggest that the homologous DUBs have diverse functions. In *Saccharomyces cerevisiae*, the *math-33* homolog *UBP15* causes mislocalization of the cell membrane protein Gap1 to cytoplasmic membranes, and results in lower permease activity [56]. In mammals, the *math-33* homolog *USP7/HAUSP* has been studied extensively for its ability to bind and deubiquitylate *p53* and the ubiquitin ligase *MDM2*, and it has also been implicated as a negative regulator of PTEN localization to the nucleus [57]–[59]. USP7 was also found to coimmunoprecipitate with the PAR-1 homolog MARK4 [60] but does not appear to be able to deubiquitylate it [61]. USP7 is also considered to be a therapeutic target for cancer therapy due to its broad role in genomic stability [62]. In contrast, there is less known about the roles of USP-46 and USP-47 in other organisms. In our study we found that the individual loss of either *usp-47* or *usp-46* have no discernible phenotypes in early *C. elegans* embryos. USP-47 has not previously been studied, but USP-46 has been shown to have deubiquitylation activity, and to regulate the levels of GLR-1 abundance in a fashion that suggests USP-46 deubiquitylation of GLR-1 on endosomes prevents degradation in the multivesicular body/lysosome pathway [63]. It is possible then that USP-46 and USP-47 may have biological roles that are redundant with other DUBs, and as a result have fewer obvious mutant phenotypes than *math-33*.

In summary, we have identified three deubiquitylase genes, *math-33*, *usp-47 and usp-46* that are required for proper polarity establishment in *C. elegans*. The enzymes encoded by these genes appear to act by stabilizing proteins that promote the interaction of the centrosome with the cell cortex. A future challenge will be to determine the targets of these deubiquitylases with respect to their role in early embryonic polarity and also whether those targets are widely conserved in animals.

## Materials and Methods

### Nematode strains

Nematode strains were maintained under standard conditions [64]. Genetic strains used in this work are listed in Table S2. The *math-33(tm3561)* mutation is a frameshift that occurs at Q508 and creates a stop codon at position 510, which is within but near the end of the UCH domain, and we confirmed this by sequencing (Figure S5A). The *math-33(ok2974)* mutation also is expected to cause a frameshift within the isopeptidase domain at or after T348 based on deletion data at Wormbase.org, sequence ID WBVar0094061. We predict that both *math-33* alleles would be null or strong loss of function alleles because the conserved enzymatic isopeptidase domain is disrupted in both mutations. We confirmed by PCR that deletions are present in strains carrying *math-33(ok2974), usp-46(ok2232)*, and *usp-47(tm4954)* at locations reported by the *C. elegans* knockout consortium and by the S. Mitani Lab. The reported endpoints are available at wormbase.org. Temperature sensitive strains were incubated at 16° for experiments in which embryonic lethality was measured. The cold sensitive *math-33(tm3561)* worms were maintained at 20° and shifted to 16° for experiments.

### Imaging and quantification

*C. elegans* embryos were released from the uterus of gravid hermaphrodites with a 10 gage needle point in distilled water or in phosphate buffered saline, pH 7. Embryos were then moved onto a 2% agarose pad on a glass slide and covered with a glass cover-slip sealed with petroleum jelly for observation. During microscopy experiments in which phenotypes were assessed, the microscope environment was cooled to 16° for the duration of the experiment, except where noted in the figure or table.

For live confocal imaging we used a Zeiss LSM 710 confocal microscope with a 63× Plan-Apochromat oil immersion lens, a stage cooled to 15°C, and Zen imaging software (Zeiss). For confocal immunostaining we used either the Zeiss LSM 710 a Leica TCS SP2 DMRE-7 microscope with a 63× oil immersion lens, and Leica imaging software. For standard wide field microscopy we used a Leica DMRA2 microscope with a 63× Leica HCX PL APO oil immersion lens, an ORCA-ER camera, and Openlab software.

Measurements were determined by measuring pixel distances using Openlab software and were converted to micrometers. Tracing of embryo borders to measure cortical domain size was performed using Openlab. To measure domain size in this way, the circumference of the embryo cortex was traced by a “walking mouse cursor” method around the embryo. PAR-2 and PAR-3 domains were measured independently; domain boundaries were defined as the point when the cortical signal equaled the background fluorescence. If one pole of an embryo was damaged during sample processing, that domain was not measured, but the opposite domain was measured and added to the average. The extent of myosin clearing was measured as a function of the entire embryo anterior-posterior length. Time-lapse confocal stacks were examined and the embryo length was measured using Image J. Then, we measured a distance through the longitudinal midline of the embryo between furthest posterior point and the edge of myosin foci clusters. Clearing was then expressed as the myosin clearing distance divided by the entire embryo length. The kymographs shown examining myosin clearing were generated with a 1 pixel-wide line through the center of the anterior-posterior axis and embryos were imaged roughly every 30 seconds. From separate 8 pixel-wide kymographs we measured flow rates as in [12] for up to three foci per embryo that remained in the posterior of the embryo during the establishment phase and were visible for at least four frames. We considered embryos to be undergoing polarity establishment after a small absence of myosin foci could be observed at the posterior. However, since some samples did not have myosin clearing, we measured flow rates for foci present during the 8 minutes prior to the transition of myosin from large foci to small puncta. We calculated flow rates by the following formula, as in [12] velocity = [Δ µm]/frames*frame rate (seconds/frame), and then converted into minutes. We observed wild type foci speeds in a range from 1.7 to 3.0 µm per minute which is different from the reported maximum of 7.9 µm per minute [12]; this difference could be due to the lower temperature (16°) at which we imaged the embryos.

Centrosome distance from the cortex was determined using Openlab software to measure the space between the edge of the centrosome to the closest edge of the cortex visible in the x-y plane of focus. Embryos were monitored and refocused at a minimum of every fifty seconds at and after the end of meiosis until the appearance of centrosomes could be detected *via* TBB-2::GFP. Afterwards, embryos were monitored over time until pseudocleavage was evident, or, in case of the absence of pseudocleavage, until after pronuclear meeting. Those centrosomes that appeared in the z plane to be very close to the bottom or the top of the embryo cortex were discarded from analysis. To measure the centrosome dwell time at the cortex we monitored the position of the centrosome relative to the closest part of the cortex over time; we defined departure from the cortex as the time that the edge of the TBB-2::GFP signal closest to the cortex was permanently more than 2 µm from the cortex.

We measured the relative area of AB compared to the area of AB plus P1 (Table 2 column 9). For this analysis we measured the circumference of blastomeres, and estimated their area from the circumference assuming a circular shaped cross-section as in [6]. We defined synchronous second cleavages of AB and P1 as the completion of P1 cytokinesis within 10 seconds of AB, and defined transverse spindles as those with spindles oriented 70° or more relative to a 0° longitudinal axis, and as those embryos in which P1 divided out of the Z plane indicating a transversely oriented mitotic spindle was present. Absence of pseudocleavage was defined as the absence of detectable persistent medial cortical invaginations in mitotic prophase. We used Openlab software (Improvision) to quantify the depth of pseudocleavage furrows. We measured the shortest distance from the deepest part of the furrow to a line drawn across the outside edges of the furrow (see Figure S3D).

### Antibody production

GST-fusion constructs fused to a fragment of MATH-33 comprised of amino acids Q517-K706 or to USP-47 amino acids E822-M1005 in pGex-6P1 vector (GE Healthcare) were used to express protein in *E. coli* for antigen production. Proteins were eluted from glutathione-agarose beads by mixing with purified GST fusion precision 3C protease (GE Healthcare) and used to produce antibodies in guinea pigs (MATH-33), (Cocalico Biologicals) or in both a guinea pig and a rabbit (USP-47), (Pocono Mountain Rabbit Farm). Affinity purification of the desired antibodies was accomplished by adsorbing sera to antigen-agarose Hi-Trap columns (GE Healthcare), and eluting the purified antibody with 1 M glycine, pH 3. Immunoflourescence signals from anti-MATH-33 and anti-USP-47 antibodies were strongly reduced in *math-33(tm3561)* and *usp-47(RNAi)* embryos respectively, demonstrating specificity.

### Immunohistochemistry

Embryos were processed by methanol fixation followed by incubations with primary and secondary antibodies [4]. Primary antibodies used were rabbit anti-PAR-2 [5], anti-PAR-3 mouse monoclonal [38], rabbit anti-NMY-2 [65], and mouse anti-Tubulin mouse monoclonal (a gift from Margaret Fuller) guinea pig anti-MATH-33, and rabbit anti-USP-47 (this study). Secondary antibodies used were Alexa Fluor 488 anti-rabbit, Alexa Fluor 488 anti-guinea pig (Invitrogen), FITC anti-rabbit, Cy3 anti-mouse, and Cy3 anti-guinea pig (Jackson Laboratories). Slides were mounted in Vectashield containing DAPI (4′,6-diamidino-2-phenylindole).

### RNA interference (RNAi)

RNAi of *math-33*, *usp-47/T05H10.1, par-4, DH11.5, pam-1, usp-46*, and *dhc-1* was performed with clones from the Ahringer library (MRC Geneservice) [66]. *DH11.5* was chosen as a control in double RNAi experiments because it is expressed embryonically according to nextDB [67] but has no detectable RNAi depletion phenotype. Depletion of 22 *C. elegans* DUBs was done with RNAi clones from both the Ahringer and Vidal RNAi libraries [66], [68]. We depleted PAR-1 by expressing the full length mRNA (GenBank Accession No. U22183) in vector pPd129.36 as in Hurd & Kemphues [69]. RNA interference of target genes was achieved by expressing dsRNA in *E. coli* followed by feeding of whole bacteria to worms [70]. dsRNA expression was induced with 1 mM IPTG at room temperature for 3 hours, and cultures were concentrated 10× before seeding non-nutrient agar plates containing 12.5 µg/ml tetracycline and 50 µg/ml carbenicillin. RNAi was performed at 16°C for 36–48 hours.

### Transgenic strains

*math-33* or *usp-47* full length cDNAs were isolated from a Gibco Proquest Library by Iproof PCR amplification (BioRad) and cloned into pIC26 (*unc-119; Ppie-1* driving a GFP-TEV-S tagged open reading frame) [71]. The *Ppie-1::gfp-tev-s::math-33 and Ppie-1::gfp-tev-s::usp-47* transgenic worm strains were generated by microparticle bombardment of *unc-119(ed4)* as in Praitis *et al.* 2001 [72].

### Phylogenetic analysis

We identified 25 UCH domain-containing proteins in *C. elegans* by using the ScanProsite tool from the ExPASy website of the Swiss Institute of Bioinformatics [73]. A phylogenetic tree showing sequence relationship of UCH domains in C. elegans as well as humans and fission yeast was generated by aligning the relevant UCH domains in Megalign (Lasergene), and by plotting node distances with Phylip software [74]. We predicted closest homologs between species by using BLAST in the species of interest (NCBI) [75]. We based our assessment of USP-47's relative conservation amongst bilatera on Treefam-generated information present on the Wormbase *usp-47* gene information webpage (wormbase.org).

## Supporting Information

Figure S1 Quantification of *math-33(tm3561)* phenotypes, and rescue of phenotypes by *Ppie-1* driven *GFP::MATH-33*. (A) The proportion of *math-33(tm3561)* worms that failed to hatch (red), arrested as larvae (orange), became sterile (purple), or became fertile adults (green). The experiment was performed at 3 different temperatures on 10 whole broods each. The number of embryos scored is given at the bottom. (B) A *Ppie-1::gfp-tev-s::math-33(itIs288)* transgene is able to rescue lethality and sterility of *math-33(tm3561)* at 16°C.
(TIF)

Figure S2 PAR-6 clearing from the posterior is reduced in *math-33(tm3561); usp-47(RNAi)*. (A) PAR-6::mCherry localization prior to establishment and after establishment. White arrowheads indicate the extent to which PAR-6 is absent from the posterior cortex. (B) Measurement of maximum PAR-6::mCherry clearing from the posterior as a proportion of the total cortex. There is less clearing of PAR-6::mCherry in *math-33(tm3561); usp-47(RNAi)* embryos, *p*<0.05, Student's t-test. The strain used in these experiments also expressed an *lgl-1::gfp* transgene.
(TIF)

Figure S3 Centrosome position in fixed embryo samples, pronuclear detachment frequency, the centrosome position in *math-33(tm3561); par-2(RNAi), and pseudocleavage examples*. (A) For the few embryos examined in Figure 3C, the average centrosome distance is significantly different than N2 controls, *p*<0.01, Student's t-test. (B) Percentage of centrosomes that were observed to be detached from a paternal pronucleus when they appeared. *pam-1* depletion causes an increase in detachment defects relative to *math-33* alone *p* = 0.042 in a Student's t-test, whereas other RNAi depletions show no significant differences. (C) *par-2(RNAi); math-33(tm3561)* embryos have an initial centrosome distance from the cortex that is not different than *math-33(tm3561)* alone. All embryos displayed pseudocleavage in this experiment.
(TIF)

Figure S4 MATH-33 and USP-47 are present in the germline. Micrographs showing oocytes and a mitotic portion of the gonad for each indicated genotype. (A) Worms immunostained for MATH-33 (red), DAPI (blue), and an overlay (red & blue). (B) Worms immunostained for USP-47 (red), DAPI (blue), and an overlay.
(TIF)

Figure S5 Phylogenetic analysis and conservation of math-33, usp-46 and usp-47. (A) Protein domain models of MATH-33, USP-46, and USP-47. Relative length of the proteins is displayed starting at amino acid 1, and the beginning and end-points of each domain are also noted. (B) An un-rooted phylogenetic tree of UCH domains aligned by amino acids. *usp-47C.e.* and *math-33C.e.* are a short distance on the tree from homologs *USP47H.s., USP7H.s., ubp5S.p.*, and *ubp15S.p.* indicating that their UCH domains share more homology than other *C.e.* UCH domains. (C) The closest homologs of the five *S. pombe* DUBs involved in asymmetry of endocytosis were used as a basis to find the next closest homologs in *C. elegans, Homo sapiens*, or *Drosophila melanogaster*. The BLAST seed sequence for each row is indicated in bold. Since *usp-47* was not the best match of any *S. pombe* sequence, we showed that *usp-47* as a seed is most similar to the *math-33* homolog *ubp5S.p.*
(TIF)

Table S1 *math-33* depletion increases embryonic lethality for some *ts* mutants not involved in polarity. *n* = the number of embryos counted for each genotype. * math-33(RNAi) lethality different from control lethality by Student's t-test, p<0.05.
(DOCX)

Table S2 Strains used in this study. * These two strains are unavailable; they were lost in an incubator malfunction.
(DOCX)

Video S1 *nmy-2::gfp* WT control. Videos S1 and S2 are time-lapse videos of confocal stacks showing NMY-2::GFP with images taken roughly every 30 seconds during the period of establishment. Evident photobleaching occurs, but large myosin foci remain visible until establishment phase when they disperse.
(MOV)

Video S2 *nmy-2::gfp*; *math-33(tm3561); usp-47(RNAi).*
(MOV)

Video S3 *tbb-2::gfp* WT control. Videos S3, S4, S5, S6 are time-lapse videos of confocal stacks using expression of TBB-2::GFP to follow the centrosomes.
(MOV)

Video S4 *tbb-2::gfp; math-33(tm3561); usp-47(RNAi)*. The centrosome appears initially on the paternal PN.
(MOV)

Video S5 *tbb-2::gfp; math-33(tm3561); usp-47(RNAi)*. The centrosome appears initially near cortex.
(MOV)

Video S6 *tbb-2::gfp; math-33(tm3561); usp-47(RNAi); rpn-10(tm1349).*
(MOV)
