# Supplementary figures and images for: Deubiquitylation Machinery Is Required for Embryonic Polarity in Caenorhabditis elegans
Source: PLoS Genet. 2012 Nov 29;8(11):e1003092. doi: 10.1371/journal.pgen.1003092 (PMC3510043; doi:10.1371/journal.pgen.1003092)

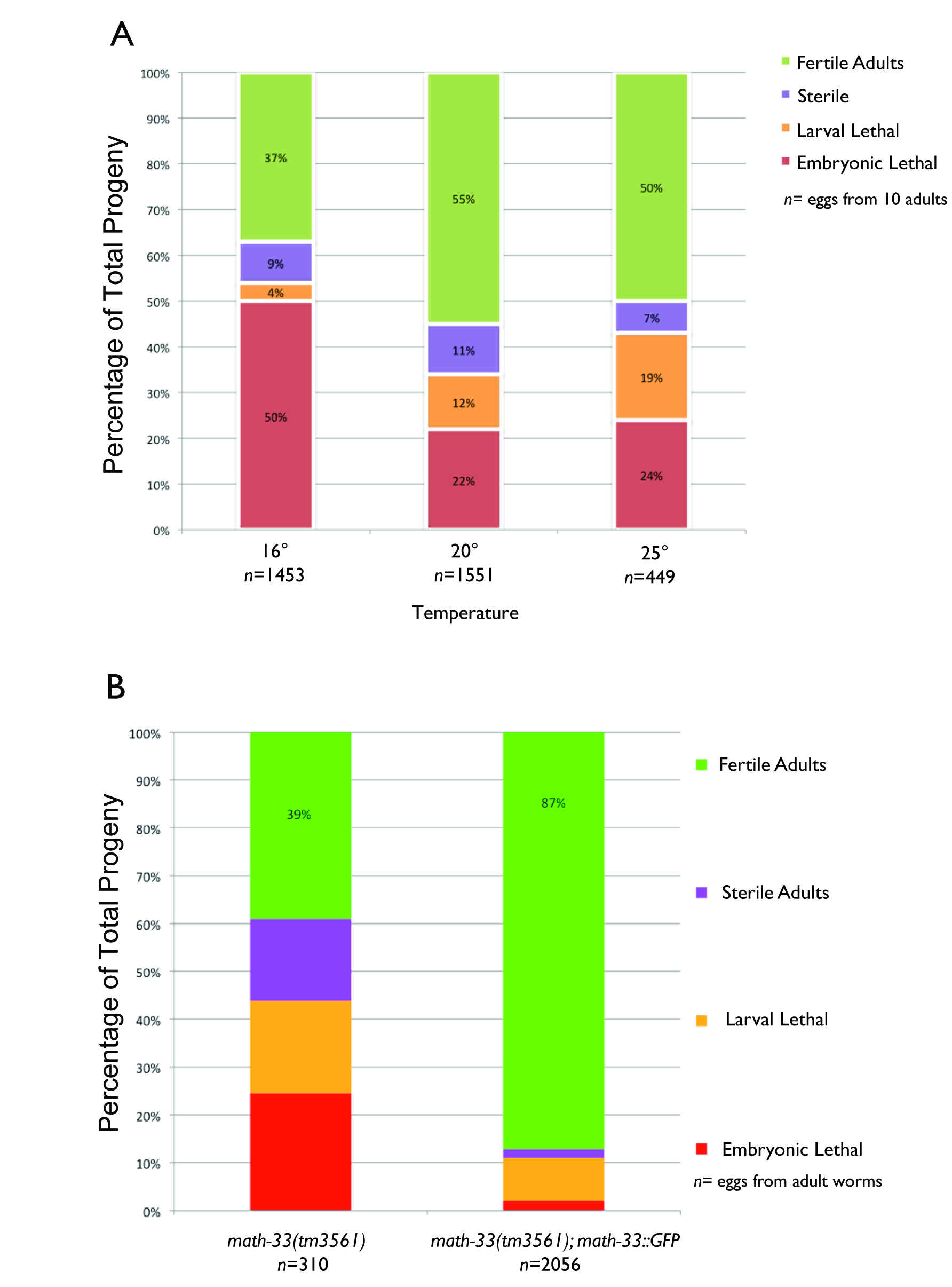

Supplement: Figure S1 — Quantification of math-33(tm3561) phenotypes, and rescue of phenotypes by Ppie-1 driven GFP::MATH-33. (A) The proportion of math-33(tm3561) worms that failed to hatch (red), arrested as larvae (orange), became sterile (purple), or became fertile adults (green). The experiment was performed at 3 different temperatures on 10 whole broods each. The number of embryos scored is given at the bottom. (B) A Ppie-1::gfp-tev-s::math-33(itIs288) transgene is able to rescue lethality and sterility of math-33(tm3561) at 16°C. (TIF) [file pgen.1003092.s001.tif]

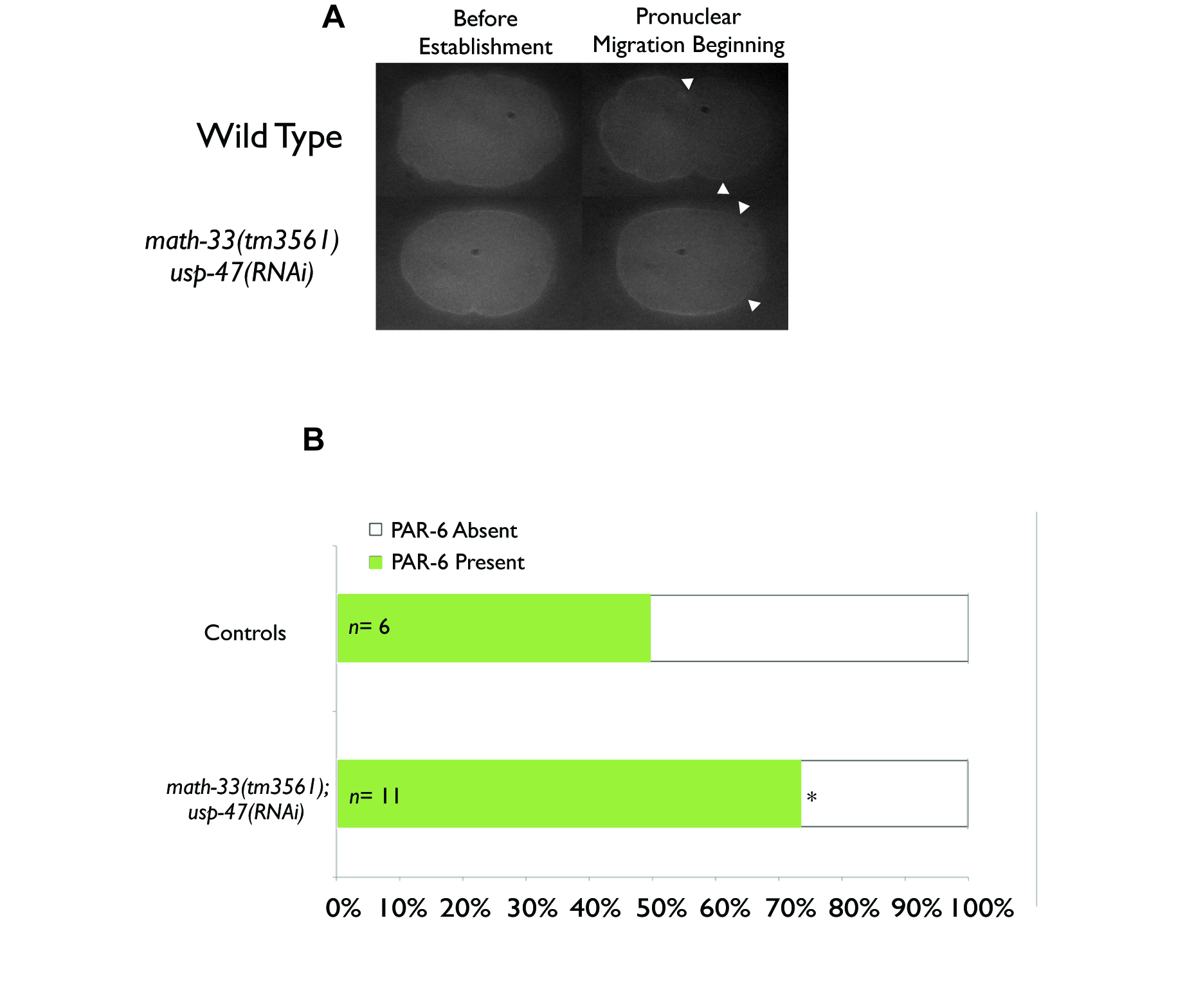

Supplement: Figure S2 — PAR-6 clearing from the posterior is reduced in math-33(tm3561); usp-47(RNAi). (A) PAR-6::mCherry localization prior to establishment and after establishment. White arrowheads indicate the extent to which PAR-6 is absent from the posterior cortex. (B) Measurement of maximum PAR-6::mCherry clearing from the posterior as a proportion of the total cortex. There is less clearing of PAR-6::mCherry in math-33(tm3561); usp-47(RNAi) embryos, p<0.05, Student's t-test. The strain used in these experiments also expressed an lgl-1::gfp transgene. (TIF) [file pgen.1003092.s002.tif]

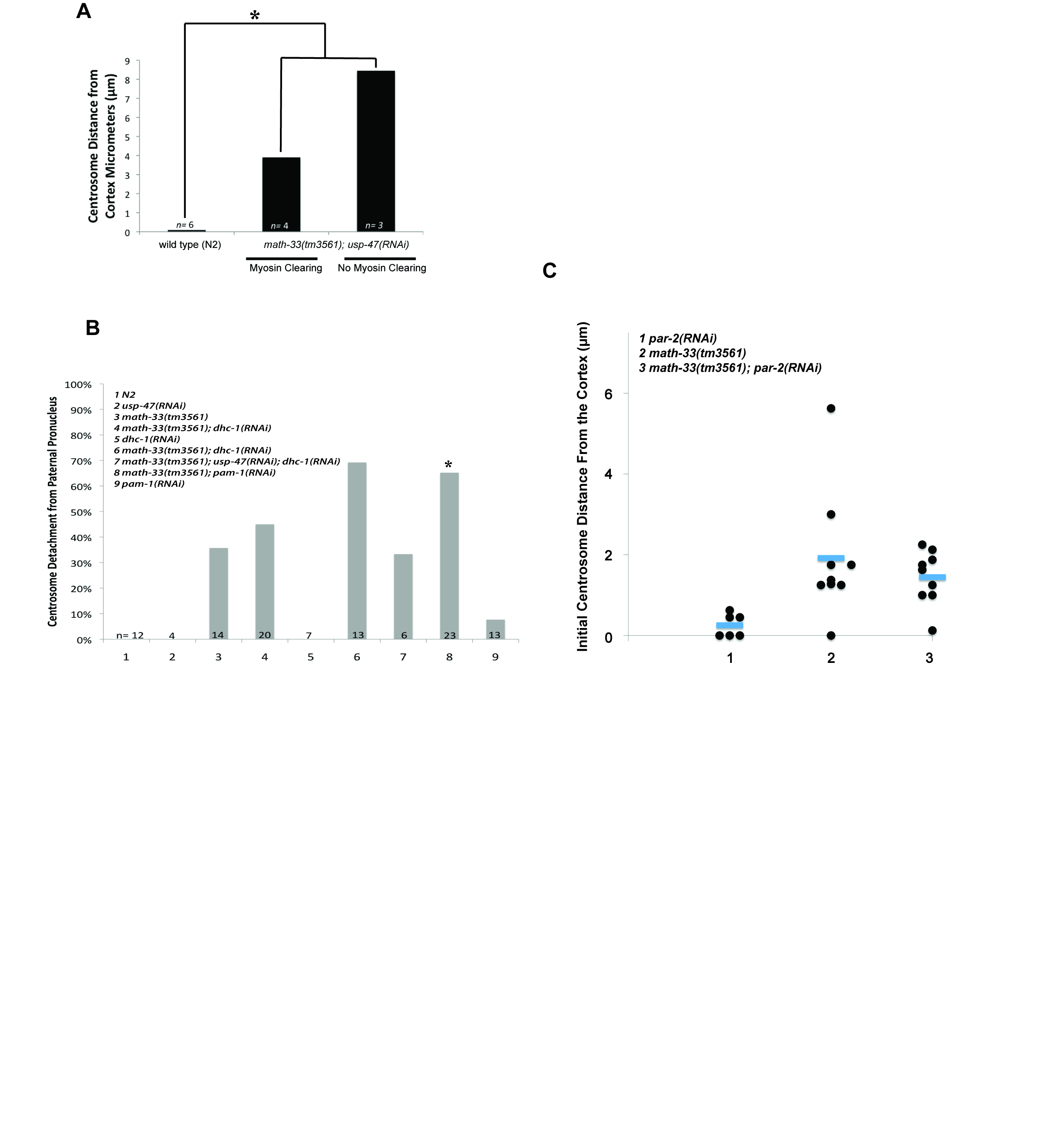

Supplement: Figure S3 — Centrosome position in fixed embryo samples, pronuclear detachment frequency, the centrosome position in math-33(tm3561); par-2(RNAi), and pseudocleavage examples. (A) For the few embryos examined in Figure 3C, the average centrosome distance is significantly different than N2 controls, p<0.01, Student's t-test. (B) Percentage of centrosomes that were observed to be detached from a paternal pronucleus when they appeared. pam-1 depletion causes an increase in detachment defects relative to math-33 alone p = 0.042 in a Student's t-test, whereas other RNAi depletions show no significant differences. (C) par-2(RNAi); math-33(tm3561) embryos have an initial centrosome distance from the cortex that is not different than math-33(tm3561) alone. All embryos displayed pseudocleavage in this experiment. (TIF) [file pgen.1003092.s003.tif]

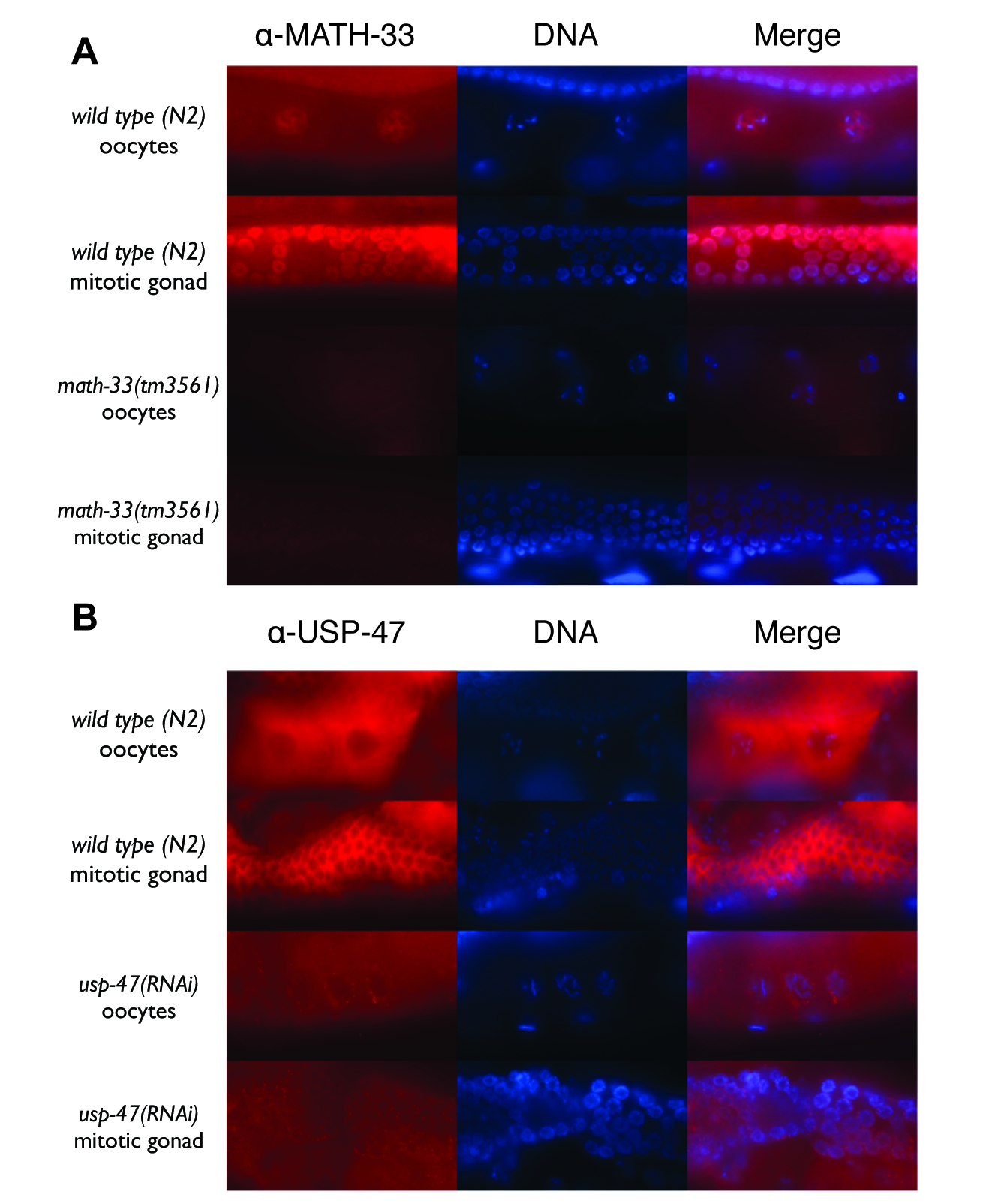

Supplement: Figure S4 — MATH-33 and USP-47 are present in the germline. Micrographs showing oocytes and a mitotic portion of the gonad for each indicated genotype. (A) Worms immunostained for MATH-33 (red), DAPI (blue), and an overlay (red & blue). (B) Worms immunostained for USP-47 (red), DAPI (blue), and an overlay. (TIF) [file pgen.1003092.s004.tif]

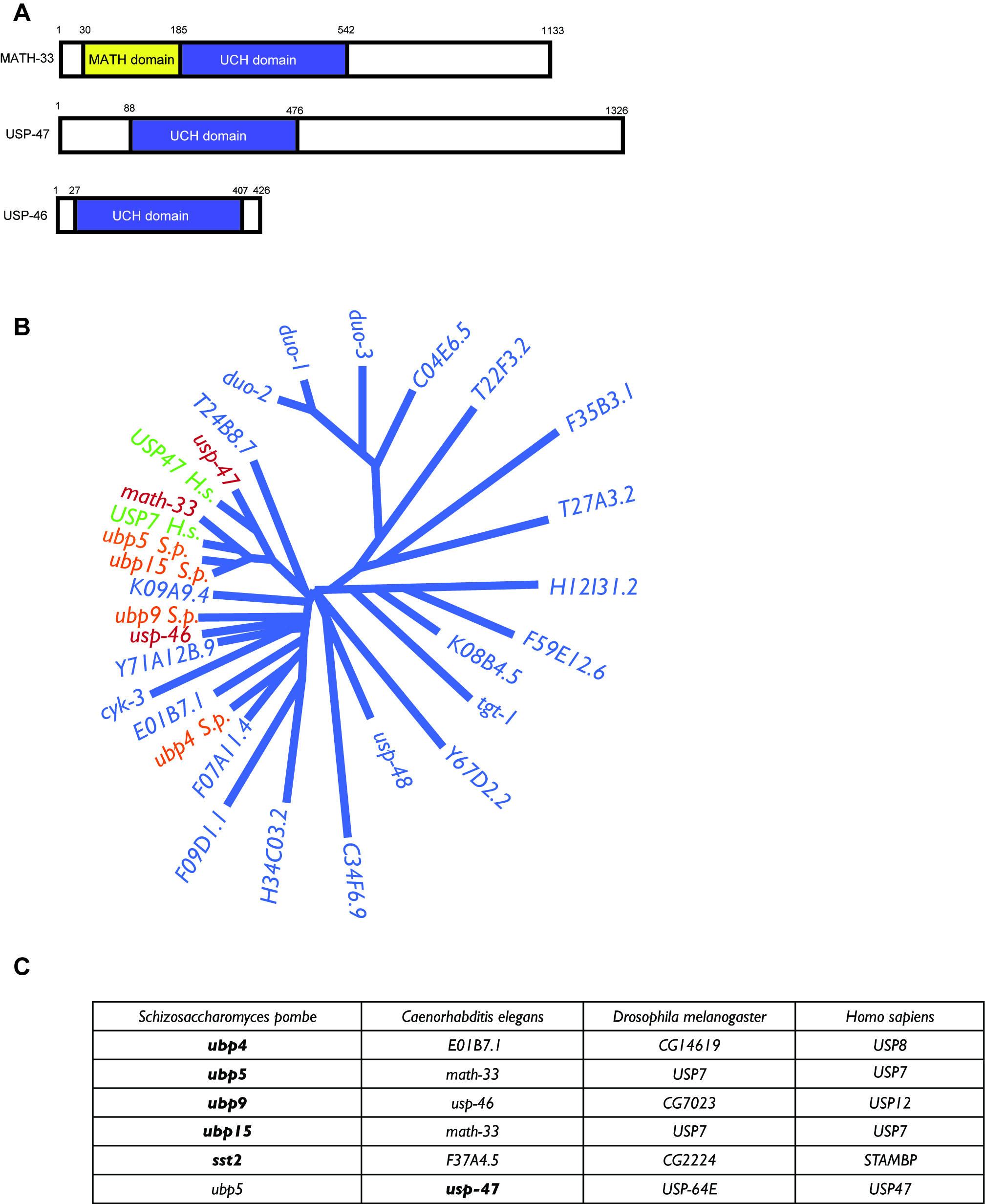

Supplement: Figure S5 — Phylogenetic analysis and conservation of math-33, usp-46 and usp-47. (A) Protein domain models of MATH-33, USP-46, and USP-47. Relative length of the proteins is displayed starting at amino acid 1, and the beginning and end-points of each domain are also noted. (B) An un-rooted phylogenetic tree of UCH domains aligned by amino acids. usp-47C.e. and math-33C.e. are a short distance on the tree from homologs USP47H.s., USP7H.s., ubp5S.p., and ubp15S.p. indicating that their UCH domains share more homology than other C.e. UCH domains. (C) The closest homologs of the five S. pombe DUBs involved in asymmetry of endocytosis were used as a basis to find the next closest homologs in C. elegans, Homo sapiens, or Drosophila melanogaster. The BLAST seed sequence for each row is indicated in bold. Since usp-47 was not the best match of any S. pombe sequence, we showed that usp-47 as a seed is most similar to the math-33 homolog ubp5S.p. (TIF) [file pgen.1003092.s005.tif]
