# Supplementary material for: Deubiquitylation Machinery Is Required for Embryonic Polarity in Caenorhabditis elegans
Source: PLoS Genet. 2012 Nov 29;8(11):e1003092. doi: 10.1371/journal.pgen.1003092 (PMC3510043; doi:10.1371/journal.pgen.1003092)
Supplement: Table S1 — math-33 depletion increases embryonic lethality for some ts mutants not involved in polarity. n = the number of embryos counted for each genotype. * math-33(RNAi) lethality different from control lethality by Student's t-test, p<0.05. (DOCX) [file pgen.1003092.s006.docx]

| Genotype | control*(RNAi)* | | *math-33 (RNAi)* | |
| --- | --- | --- | --- | --- |
|  | Embryo lethality | *n* | Embryo lethality | *n* |
| N2 – wild type | 1% | 877 | 6% | 525 |
| *emb-9(g23ts)* | 0% | 1746 | 20%* | 1136 |
| *emb-9(b189ts)* | 2% | 1698 | 4% | 2014 |
| *zyg-9(b288ts); unc-4(e120)* | 9% | 977 | 49%* | 911 |
| *mom-2(ne874ts); unc-5(e53)* | 3% | 253 | 2% | 411 |
| *mom-4(ne1539ts)* | 0% | 1086 | 1% | 1056 |
| *wrm-1(ne1982ts)* | 1% | 754 | 2% | 1201 |
| *mig-5(rh147ts)* | 5% | 613 | 10% | 645 |
| *lit-1(ne1991ts)* | 11% | 540 | 15% | 818 |

**Table S1. *math-33* depletion increases embryonic lethality for some *ts* mutants not involved in polarity.***n=* the number of embryos counted for each genotype.

* *math-33(RNAi)* lethality different from control lethality by Student’s t-test, p<0.05.
