# Supplementary material for: Deubiquitylation Machinery Is Required for Embryonic Polarity in Caenorhabditis elegans
Source: PLoS Genet. 2012 Nov 29;8(11):e1003092. doi: 10.1371/journal.pgen.1003092 (PMC3510043; doi:10.1371/journal.pgen.1003092)
Supplement: Table S2 — Strains used in this study. * These two strains are unavailable; they were lost in an incubator malfunction. (DOCX) [file pgen.1003092.s007.docx]

**Table S2. Strains used for this study.**

| N2 | wild type |
| --- | --- |
| AZ235 | *unc-119(ed3) III; ruls48[pAZ147 Ppie-1::tbb-2::gfp, unc-119(+)]* [1] |
| KK1181 | *usp-47(tm4954) II* outcrossed to N2 one time |
| FX2616 | *lgl-1(tm2616) X* [2] |
| JJ1473 | *unc-119(ed3) III; zuls45[nmy-2::nmy-2::gfp+unc-119(+)] V*. [3] |
| KK83 | *par-2(e2030) unc-32(e189) III* [4] |
| KK196 | *par-3(e2074)III; sup-7(st5) X* [4] |
| KK300 | *par-4(it57ts) V* [5] |
| KK574 | *par-2(it87) unc-32(e189) III* [5] |
| KK747 | *par-2(lw32) unc-45(e286ts)/ sC1 III* [6] |
| KK810 | *par-1(zu310ts) V* |
| KK879 | *itIs153 [Ppie-1::par-2::gfp] II; par-2(lw32) unc-45(e286ts) III* |
| KK1033 | *par-2(it5ts) III* – outcrossed to N2 one time from KK418 [4] |
| KK1042 | *itIs272[Ppar-6::par-6::mCherry, unc-119(+); unc-119(ed4) III* (K. Basch, H. Kim, K. Kemphues unpublished) |
| KK1056 | *math-33(tm3561) V* – outcrossed to N2 nine times from RB2194 |
| KK1064 | *itIs288[Ppie-1::gfp-tev-s::math-33 +unc-119(+)]; unc-119(ed4) III* |
| KK1069 | *math-33(tm3561) V / nT1[qIs51] myo-2::gfp pes-10::gfp F22B7.9::gfp (IV:V)* |
| KK1070 | *itIs153[Ppie-1::par-2::gfp] II; math-33(tm3561) V / nT1[qIs51] myo-2::gfp pes-10::gfp F22B7.9::gfp (IV:V)* |
| KK1091 | *unc-119(ed4) III; math-33(tm3561)V; itIs288[Ppie-1::gfp::math-33, unc-119(+)]* |
| KK1094 | *unc-119(ed3) III; math-33(tm3561)V; ruls48[pAZ147 Ppie-1::tbb-2::gfp, unc-119(+)]* |
| KK1098 | *math-33(tm3561)* *zuls45[Pnmy-2::nmy-2::GFP, unc-119(+)] V* |
| KK1119 | *par-2(lw32) unc-45(e286ts) III; itIs256[Plgl-1::lgl-1::gfp, unc-119(+)] V* [2] |
| KK1103 | *math-33(tm3561) V; itIs256[Plgl-1::lgl-1::gfp, unc-119(+)]; itIs272[Ppar-6::par-6::mCherry, unc-119(+)]* |
| KK1136 | *itIs290[Ppie-1::gfp-tev-s::usp-47 +unc-119(+)]; unc-119(ed4) III* |
| KK1138 | *rpn-10(tm1180) I; math-33(tm3561) V* |
| KK1139 | *rpn-10(tm1349) I; math-33(tm3561) V* |
| KK1174 | *rpn-10(tm1349) I;* *math-33(tm3561)V; ruls48[pAZ147 Ppie-1::tbb-2::gfp, unc-119(+)]* |
| KK1180 | *usp-46(ok2232) III* – outcrossed to N2 three times * |
| KK1181 | *usp-46(ok2232) III; math-33(tm3561) V / nT1[qIs51] myo-2::gfp pes-10::gfp F22B7.9::gfp (IV:V)** |
| RB2194 | *math-33(ok2974) V* |
| UM25 | *rpn-10(tm1349) I* [7] |
| UM26 | *rpn-10(tm1180) I* [7] |

* These two strains were lost due to incubator malfunction.

**References:**

1. Praitis V, Casey E, Collar D, Austin J (2001) Creation of low-copy integrated transgenic lines in Caenorhabditis elegans. Genetics 157: 1217–1226. Available:http://www.pubmedcentral.nih.gov/articlerender.fcgi?artid=1461581&tool=pmcentrez&rendertype=abstract.

2. Beatty A, Morton D, Kemphues K (2010) The C. elegans homolog of Drosophila Lethal giant larvae functions redundantly with PAR-2 to maintain polarity in the early embryo. Development 137: 3995–4004. Available:http://www.pubmedcentral.nih.gov/articlerender.fcgi?artid=2976283&tool=pmcentrez&rendertype=abstract. Accessed 29 March 2012.

3. Nance J, Munro EM, Priess JR (2003) C. elegans PAR-3 and PAR-6 are required for apicobasal asymmetries associated with cell adhesion and gastrulation. Development 130: 5339–5350. Available:http://www.ncbi.nlm.nih.gov/pubmed/13129846. Accessed 2 March 2012.

4. Kemphues KJ, Priess JR, Morton DG, Cheng NS (1988) Identification of genes required for cytoplasmic localization in early C. elegans embryos. Cell 52: 311–320. Available:http://www.ncbi.nlm.nih.gov/pubmed/3345562.

5. Morton DG, Roos JM, Kemphues KJ (1992) par-4, a Gene Required for Cytoplasmic Localization and Determination of Specific Cell Types in Caenorhabditis elegans Embryogenesis. Genetics 130: 771–790.

6. Levitan DJ, Boyd L, Mello CC, Kemphues KJ, Stinchcomb DT (1994) par-2, a gene required for blastomere asymmetry in Caenorhabditis elegans, encodes zinc-finger and ATP-binding motifs. Proceedings of the National Academy of Sciences of the United States of America 91: 6108–6112. Available:http://www.pubmedcentral.nih.gov/articlerender.fcgi?artid=44147&tool=pmcentrez&rendertype=abstract.

7. Labbé J-C, Pacquelet A, Marty T, Gotta M (2006) A genomewide screen for suppressors of par-2 uncovers potential regulators of PAR protein-dependent cell polarity in Caenorhabditis elegans. Genetics 174: 285–295. Available:http://www.pubmedcentral.nih.gov/articlerender.fcgi?artid=1569778&tool=pmcentrez&rendertype=abstract. Accessed 23 April 2012.
